# Mycotoxins Affecting Animals, Foods, Humans, and Plants: Types, Occurrence, Toxicities, Action Mechanisms, Prevention, and Detoxification Strategies—A Revisit

**DOI:** 10.3390/foods10061279

**Published:** 2021-06-03

**Authors:** Chinaza Godswill Awuchi, Erick Nyakundi Ondari, Chukwuka U. Ogbonna, Anjani K. Upadhyay, Katarzyna Baran, Charles Odilichukwu R. Okpala, Małgorzata Korzeniowska, Raquel P. F. Guiné

**Affiliations:** 1 Department of Biochemistry, Kampala International University, Bushenyi P.O. Box 20000, Uganda; drerickondarin@gmail.com; 2 School of Natural and Applied Sciences, Kampala International University, Kampala P.O. Box 20000, Uganda; 3 Department of Biochemistry, Federal University of Agriculture Abeokuta, Abeokuta P.M.B. 2240, Ogun State, Nigeria; ogbonnacu@funaab.edu.ng; 4 School of Biotechnology, KIIT University, Bhubaneswar 751019, Odisha, India; upadhyayanjanikumar6@gmail.com; 5 Faculty of Biotechnology and Food Sciences, Wrocław University of Environmental and Life Sciences, 51-630 Wrocław, Poland; katarzynapsp@vp.pl (K.B.); malgorzata.korzeniowska@upwr.edu.pl (M.K.); 6 CERNAS Research Centre, Polytechnic Institute of Viseu, 3504-510 Viseu, Portugal

**Keywords:** mycotoxins, food products, action mechanisms, toxicity challenges, detoxification, prevention strategies

## Abstract

Mycotoxins are produced by fungi and are known to be toxic to humans and animals. Common mycotoxins include aflatoxins, ochratoxins, zearalenone, patulin, sterigmatocystin, citrinin, ergot alkaloids, deoxynivalenol, fumonisins, trichothecenes, *Alternaria* toxins, tremorgenic mycotoxins, fusarins, 3-nitropropionic acid, cyclochlorotine, sporidesmin, etc. These mycotoxins can pose several health risks to both animals and humans, including death. As several mycotoxins simultaneously occur in nature, especially in foods and feeds, the detoxification and/or total removal of mycotoxins remains challenging. Moreover, given that the volume of scientific literature regarding mycotoxins is steadily on the rise, there is need for continuous synthesis of the body of knowledge. To supplement existing information, knowledge of mycotoxins affecting animals, foods, humans, and plants, with more focus on types, toxicity, and prevention measures, including strategies employed in detoxification and removal, were revisited in this work. Our synthesis revealed that mycotoxin decontamination, control, and detoxification strategies cut across pre-and post-harvest preventive measures. In particular, pre-harvest measures can include good agricultural practices, fertilization/irrigation, crop rotation, using resistant varieties of crops, avoiding insect damage, early harvesting, maintaining adequate humidity, and removing debris from the preceding harvests. On the other hand, post-harvest measures can include processing, chemical, biological, and physical measures. Additionally, chemical-based methods and other emerging strategies for mycotoxin detoxification can involve the usage of chitosan, ozone, nanoparticles, and plant extracts.

## 1. Introduction

Mycotoxins are among the secondary metabolites released by molds, particularly fungi, which contaminate agricultural products pre-harvest, during harvest, and/or post-harvest and mostly exhibit toxicity to animals and humans [1,2,3]. Common mycotoxins include aflatoxins, ochratoxins, zearalenone (ZEA), patulin, sterigmatocystins (STCs), citrinin, ergotamine, deoxynivalenol (DON), fumonisins, trichothecenes, etc. Human exposure to these mycotoxins can occur via inhalation, ingestion, or contact, possibly due to contamination (of foodstuffs) within the food supply chain [4,5]. In times of historical floods, wars, and famine, mycotoxins have led to toxic outbreaks of epic proportions that devastated the human race [4]. Mycotoxins are also considered as emerging toxic pollutants [6], attracting global attention as significant contaminants that diversely affect both animal and human health, which also increases the economic burden across the food supply chain [1,7]. Prevention is one of the significant strategies that can help fight mycotoxins, which needs to be applied before harvesting, in processed foods, and even in raw materials.

More than 500 mycotoxins have been reported, most of which are under regulation or testing, while new mycotoxins are often discovered [8,9,10]. Plant metabolisms may release “hidden mycotoxins” that are modified substances that might not be detected with the methods of analysis used to identify their parent compounds [3,10]. The mycotoxins people get exposed to and the problems that arise may be due to the exposure differences from one country to another. In some countries in Africa and Asia, mycotoxin exposures are greatly associated with the overreliance on large quantities of crops that are prone to mycotoxin infection, including maize and peanut crops. In such cases, exposure to mycotoxins could actually exceed safe levels, even with low contamination levels [4]. Children are the most sensitive and vulnerable to mycotoxins’ toxic effects due to their heightened sensitivity to immunological, nervous, endocrine, and neurotoxic effects as well as their greater overall exposure when considering body mass, which can completely differ from adults [11]. By studying mycotoxins’ involvement in the environmental enteropathy pathogenesis, which is not sufficiently understood, the subclinical condition could manifest, for example, as decreased intestinal resorptive capacities, which is likely to be associated with stunting in children. Knowledge of this may offer strategies for improving growth in children [12] by providing information sufficient enough to understand the underlying mechanisms and possible ways to avoid it.

Some mycotoxins have been applied in clinical medicines. For instance, ergotamine has been applied for the treatment of vascular headaches; ergotism cases have been reported when used in combination with some antibiotics, including tetracycline and erythromycin, or with cytochrome P450 inhibitors, including HIV protease inhibitors [13,14]. For a better understanding of mycotoxin biology, it is important to acknowledge that the predominantly affected systems or organs may differ significantly across different species of animals. With this in mind, it is a bit challenging to fully understand the molecular pathways associated with the pathogeneses that emerge after mycotoxin infection. For instance, fumonisins cause esophageal cancer in humans, hepatotoxicity and nephrotoxicity in rodents, equine leukoencephalomalacia in horses, and severe pulmonary edema, left ventricular dysfunction, and hepatotoxicity in pigs [3,15,16]. Mycotoxins usually co-occur in agricultural commodities. Some fungi can release two or more mycotoxins. *Fusarium* species can produce ZEA, trichothecenes, and fumonisins [10,17]. Whereas biological effects have been generally studied individually, mycotoxin exposure often concomitantly occurs with several mycotoxins that might interact. Ochratoxin A (OTA) can act along with aflatoxin B_1_, penicillic acid, citrinin, or fumonisin B_1_; although exposures to mycotoxin co-contaminations have received relatively less attention in comparison with exposures to individual mycotoxins [18].

Multiple factors interact in the pathogenesis of mycotoxicosis (illness caused by mycotoxins) and can include genetic, physiological, and environmental aspects. Such factors specific to mycotoxins, considered often problematic, do shape the metabolism and toxicity that confirm exposure and diagnoses. As several mycotoxins simultaneously occur in nature, especially in foods and feeds, the detoxification and/or total removal of mycotoxins remain challenging. Moreover, given that the volume of scientific literature regarding mycotoxins is steadily on the rise, there is need for continuous synthesis of the body of knowledge. To supplement existing information, knowledge of mycotoxins affecting animals, foods, humans, and plants, with more focus on types, toxicity, and prevention measures, including strategies employed in detoxification and removal, were revisited in this work.

## 2. Major Groups of Mycotoxins: Occurrence, Production, and Toxicities

**Figure 1:**
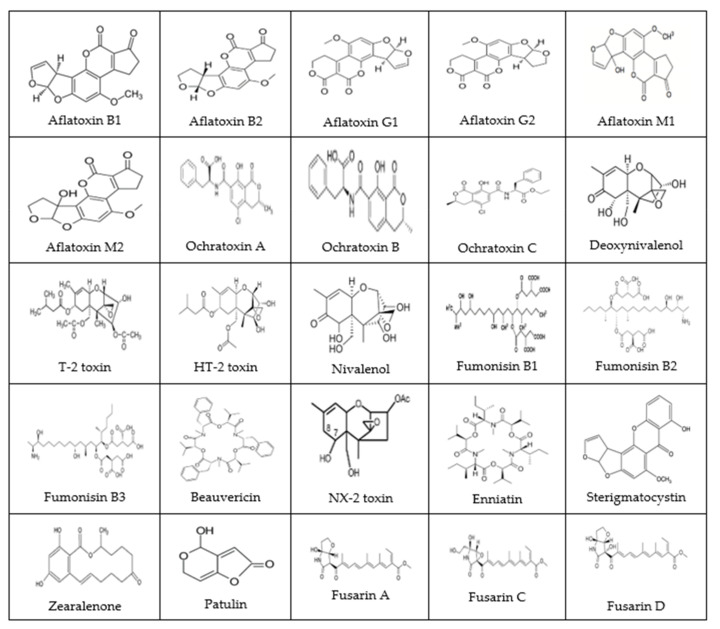
The chemical structures of common mycotoxins (Reprinted/Adapted from sources [1,3,7,9,19]).

In this section, we look at major groups of mycotoxins, from aflatoxins to other common mycotoxins like fusarins, etc., and attempt to describe the occurrence, production, and toxicities of each. The chemical structures of common mycotoxins are shown in Figure 1. Major common mycotoxins, their (established/evolving) toxicities, and maximum allowable limits and associated remarks are shown in Table 1.

### 2.1. Aflatoxins

Aflatoxins are a group of mycotoxins primarily produced by *Aspergillus flavus*, *A. bombycis*, *A. pseudotamarii*, *A. nomius*, and *A. parasiticus*, and can infest several crops, foods, and agricultural products [19,20]. Aflatoxins gained significance in 1960s in the turkey “X” disease epidemic that caused deaths and severe hepatic lesions in turkeys, chickens, and ducks fed with mold-infested peanut meal [21,22]. Aflatoxins consist of twenty related polycyclic structures belonging to a class of compounds known as the furanocoumarins. *A. flavus*, an opportunistic pathogen, mostly thrives in oilseed crops, including tree nuts, cotton, peanuts, maize, etc. [20,23]. *A. flavus* is present as mycelia in plant tissues and as sclerotia or conidia in soil, and usually occurs in warm climates with latitudes of 16 to 35 degrees; it is not common in latitudes above 45 degrees [20]. Cottonseed storage lipids, especially triglycerides, have been shown to support the production of aflatoxin B1 (AFB1). Following the removal of lipids from cottonseed, the production of aflatoxins was reduced by at least 800-fold; reconstituting the seeds using cottonseed lipids resulted in the production of mycotoxins to initial levels [24]. While *A. parasiticus* only infects ground crops, *A. flavus* infects several plants [20]. Quantitative and qualitative differences in aflatoxin production capabilities of several strains of molds have been studied. Roughly half of the strains of *A. flavus* can produce aflatoxins [25]. *A. sojae* and *A. oryzae,* used in making miso, sake, and soy sauce, closely relate to *Aspergillus parasiticus* and *A. flavus*, and comprise homologs of biosynthetic genes of many aflatoxins but have not been reported to make aflatoxins [25].

Aflatoxins are the most significant mycotoxins with regards to their occurrence, human impact, toxicity, and abundance [2]. The four major groups of aflatoxins include aflatoxin B1 (AFB1), aflatoxin B2 (AFB2), aflatoxin G1 (AFG1), and aflatoxin G2 (AFG2). In aflatoxin names, “2” shows that its structural isomer is missing a double bond in comparison with aflatoxins with a corresponding “1” [26]. Names of the four main aflatoxins related to foods are based on their green (G) or blue (B) fluorescence in ultraviolet light as well as their chromatographic mobilities [27]. Two more aflatoxins, aflatoxin M1 (AFM1) and aflatoxin M2 (AFM2), are not associated with cereals but can be detected in milk of mammals that feed on a diet infested with AFB1 and AFB2 and are their metabolic products. Toxicity levels are reduced in the order AFB1, AFB2, AFG1, and AFG2 [28]; AFB1 has the most toxicity compared to other aflatoxins and is associated with hepatocellular carcinoma [2,3,26].

A fungus may produce two or more aflatoxins. For example, *Aspergillus parasiticus* produces AFB1, AFB2, AFG1, and AFG2, and as a result these aflatoxins are usually found as mixtures in foods. *Aspergillus flavus* is morphologically grouped into the S strain, with diameter of <400 μm sclerotia, and the L strain, with diameter of >400 μm sclerotia [20]. The two strains produce AFB1 and AFB2; the S strain can also produce AFG1 and AFG2 [20]. Through polluted air and foods, aflatoxins were estimated have impacts on over 5 billion individuals in regions with humid and warm climate conditions such as the tropical and subtropical regions, and their occurrence is more common in regions with poor food storage and drying methods [29,30]. A large segment of certain populations, such as sub-Saharan Africa and southern China, face regular exposure to aflatoxins, even beginning from intrauterine periods, and exposure could last throughout the lifespan of the individual [31]. The determination of exposure to aflatoxins from foods can pose challenge mostly due to the numerous food items that usually contain aflatoxins and also the challenges in estimating exact individuals’ food consumption patterns [1,31]. We often eat a combination of foods. The biomarkers for exposure to aflatoxins are more useful; one of the assays involves the measurement of the amount of aflatoxins that is bound to albumin. Studies involving rodents showed that the adducts of AFB1-albumin form in dose-dependent manners, showing adduct formation between liver DNA and AFB1 [1]. Estimating aflatoxin–albumin adduct levels showed how exposures vary seasonally. Adduct levels in children in Gambia have been reported to be significantly higher in May compared to November, possibly showing exposure to (or consumption of) stored crops [1]. The target of the DNA of most activated forms of AFB1 is the guanine N^7^ atom that is located in the DNA’s major groove and can be accessed for reactions. The AFB1—N^7^-guanine adducts are among the most reliable and informative urinary biomarkers, although they only show recent exposure [25,32]. An AFB1-formamidopyrimidine adduct in rats has been reported to be the second DNA adduct that is most abundant [33].

Aflatoxins are stable in heat with mutagenic and teratogenic effects. Aflatoxins are strong carcinogens in humans; also, their carcinogenicity can occur in birds, rodents, nonhuman primates, etc. [27,30]. After consumption, AFB1 and AFB2 metabolize to AFM1 and AFM2, respectively [28]. AFB1 conversion to AFM1 is carried out through hydroxylating the difuranocoumarin ring’s tertiary carbon, and the -OH group promotes the solubility in water, which allows quick excretion in feces, urine, and milk [19]. Studies involving the use of animal models showed that roughly 6% of AFB1 can be metabolized and secreted as AFM1 in milk, however, the transformation rates vary depending on animals and with numerous factors, such as animal health, digestion rate, diet, etc. [19]. AFM1 contamination of milk has been seen in milk and dairy products globally, and is based on several factors, such as seasons, farming systems’ diversity, geographic location, and environmental conditions. Many studies reported that milk and dairy products produced in warm seasons had less contamination than those produced in cold seasons, likely due to the conditions favorable for the growth of fungi in cattle feed stored for prolonged periods of time in cold seasons [19]. In dairy animals’ milk, AFM1 is detectable within 12 h after the animals consume feed contaminated with AFB1 [34]. AFM1 exposure in humans usually occurs via milk exposure. The AFM1 maximum residue level permitted in milk has been set by the European Union and the United States at 50 ng/kg and 500 ng/kg of raw milk, respectively. For the avoidance of carryover, the AFB1 maximum residue level permitted in feeds of lactating cows is set at 5 μg AFB1/kg, 10 μg/kg, and 20 μg/kg of feeds in the EU, in China, and in the US, respectively [34].

Aflatoxin contamination of crops can occur pre-harvest due to heat- or drought-caused stress in the plants’ reproductive stage [25,35]. Post-harvest contamination of crops poses a significant challenge and is usually linked to inappropriate conditions of storage, including insect activity and excess moisture [20]. Damage by insects is associated with aflatoxin presence in crops, probably due to the damage in plants that allows for the entry of fungi [23]. Other factors that increase the production of aflatoxins can be considered as stressors. Examples of stressors include plant diseases, excess plant density, competition from weeds, plant oxidative stress, and insufficient plant nutrition [35,36]. The biosynthesis of aflatoxins is optimum within the temperature range 28 °C to 35 °C, although it can be inhibited at temperatures above 36 °C [27,37]. *A. flavus* genes’ genome-wide expression could reduce with an increase in temperature from 28–37 °C [37]. The most researched nutritional factors that affect aflatoxin production include those associated with carbon and nitrogen sources [38,39,40,41,42,43,44,45,46,47]. Moreover, simple sugars, including glucose, maltose, and fructose, can support aflatoxin production, whereas complex sugars, including lactose, can inhibit aflatoxin production [27]. Reduced nitrogen availability, acidic pH (approximately 4.5), temperatures below 35 °C, and oxidative stress are conducive to aflatoxin biosynthesis, whereas basic pH (approximately 8), temperatures above 36 °C, antioxidant presence, and oxidized nitrogen sources are not conducive for aflatoxin biosynthesis [48]. The production of AFB1 and AFB2 by *A. flavus* would respectively increase tyrosine and reduce tryptophan. On the other hand, tryptophan increases with the production of AFB1 and AFG1, while tyrosine reduces the production of AFG1 but increases the production of AFB1 and AFB2 by *A. parasiticus* [49].

There are conspicuous interspecies variations in the vulnerability to carcinogenesis resulting from exposure to AFB1, with mice being the most resistant and rats being the most susceptible [50]. The mice relative resistance could be because of the highly constitutive expressions in the murine liver of the glutathione S-transferase A3 subunit (mGSTA3), which is absent in humans [51]. Many kinds of aflatoxicosis have been reported in humans after exposure to aflatoxins. Acute aflatoxicosis caused by one or more exposures can lead to death in some severe cases, whereas chronic aflatoxicosis may result in hepatocellular carcinoma, suppression of the immune system, and stunted growth [20,23]. A massive outbreak with high rates of mortality took place in over 200 villages in western India in 1974. Those affected presented with portal hypertension, rapidly developing ascites, and jaundice. The outbreak, which occurred simultaneously in all the villages, contended against etiology of infectious disease. Consumption of maize highly infested with *Aspergillus flavus* was reported to be the cause. Studies of contaminated samples indicated that those affected may have eaten aflatoxins at levels of 2 to 6 mg daily for up to a month [52].

In 1988, thirteen Chinese children lost their lives due to acute hepatic encephalopathy, which resulted from an outbreak in a city in northwestern Malaysia called Perak. Epidemiological studies showed those affected had consumed Chinese noodles hours prior to their death. Those affected were dispersed geographically across six towns in two districts on the way to where the factory-supplied noodles were distributed. The postmortem studies confirmed the presence of aflatoxins [53]. In April 2004 there was a huge outbreak in Kenya, which was among the largest outbreaks of aflatoxicosis in history, causing at least 125 deaths and 317 cases. This specific outbreak was associated with homegrown maize that had been contaminated with aflatoxins [54]. Acute aflatoxicosis has been reported in both humans and animals. Studies showed that two-thirds of 600 feeder pigs lost their lives following exposure to between 2500 and 3500 μg of aflatoxins per kilogram in feed from draught-stressed *A. flavus*-contaminated maize stored in conditions that favored the production of mycotoxins [55]. Hepatic failures and deaths have been reported in dogs after consuming aflatoxin-contaminated commercial dog foods; illness was described in horses after consuming corn contaminated with aflatoxins [56,57]. In humans, chronic exposure to AFB1 was associated with hepatocellular carcinoma. Indeed, AFB1 remains among the most powerful compounds able to cause hepatocellular carcinoma in humans and is largely considered a potent carcinogen [29,30]. The International Agency of Research on Cancer (IARC) classified AFB1, AFB2, AFG1, and AFG2 into group 1, which includes substances with sufficient evidence to support their carcinogenicity in humans [26,58]. In addition, as immunosuppressants, aflatoxins can induce immunosuppression [29].

In animals and humans, the metabolite of aflatoxins responsible for their carcinogenic properties is the short-lived AFB-2,3-epoxide, currently known as AFB1-8,9-epoxide (AFBO), which has the capacity to form adducts with DNA and proteins and results in mutations [29,59]. Cytochromes P-450 3A4 and 1A2 are major liver enzymes that are responsible for converting AFB1 to AFBO. Glutathione S-transferase (GST), the detoxifying enzyme that catalyzes AFBO conjugation with glutathione, provides protection against the liver-damaging effects [29]. A main AFBO detoxification pathway is via its enzymatic conjugation with glutathione S-transferase, and vulnerability to liver carcinogenesis in many rodent species due to exposure is inversely proportional to levels of glutathione S-transferase [60]. Additionally, the AFB1 mutational effects that are widely studied involve the p53 gene in humans. Approximately half of individuals with hepatocellular carcinoma residing in areas with a risk of exposure to aflatoxins harbor mutations in the p53 gene [61,62], which would vary by their nature as well as position [63]. Approximately 50% individuals that show hepatocellular carcinoma following exposure to aflatoxins are believed to have G–T transversions in p53 gene clusters at codon 249 from exon 7, which substitute the residue of arginine with serine [63]. The use of rat microsome-activated AFB1 in human hepatocarcinoma cells to assess mutagenesis in codons 247–250 revealed that mutations could occur in many codons, with G to T transversions situated in the codon 249 third position [62]. This hotspot of mutation is among the six most common cancer-linked mutations in the p53 gene [64]. The protein of the mutant p53 stimulates the growth of hepatocyte and obstructs p53-mediated transcriptions as well as apoptosis [63]. These results explain the p53 mutations’ involvement in the selectivity of hepatocytes clonal expansion as a result of exposure to aflatoxins [65].

Synergistic interactions between AFB1 and chronic infection of hepatitis B have been reported, and a number of potential mechanisms have been explained. Examples of such mechanisms include: (a) the hepatitis B virus’s ability to cause the formation of mutagenic intermediate by cytochrome P450; (b) the hepatitis B virus’s ability to obstruct the repair of nucleotide excision; and (c) the hepatocyte damage induced by the virus that eventually increases the mutation possibility [66]. Another study investigated 18,244 Chinese men and showed that exposure to aflatoxins, evaluated through detecting urinary metabolites, increased hepatocellular carcinoma risk by approximately twofold, antigens of hepatitis B increased it by approximately fivefold, and joint exposure increased it by approximately 60-fold [67]. Synergistic interactions with the hepatitis C virus were reported, although the relationship is not yet fully understood like that of the hepatitis B virus [68]. Risk assessment analysis showed that around 25% of global cases of hepatocellular carcinoma can be associated with aflatoxin exposure, which commonly occurs in places with high hepatitis B prevalence, such as China, Southeast Asia, and sub-Saharan Africa [69]. Aflatoxins in humans being associated with cancer in organs, including the lungs, has been proven using animal models, demonstrating lung carcinogenesis due to exposure to aflatoxins [70,71,72]. This is often not through ingestion but via a respiratory route.

All species of *Aspergillus* known so far have eight chromosomes. In 2005, the complete genome of *Aspergillus flavus* was released. Like *Aspergillus oryzae*, the genome size of *Aspergillus flavus* is around 37 Mbp, somewhat bigger than the approximate 30-Mbp *Aspergillus fumigatus* genome size, and it encodes more than twelve thousand functional genes [20,73,74,75,76]. Biosynthesis of aflatoxins is believed to require not less than 23 biochemical reactions; genes involved in these biochemical reactions have been annotated and sequenced [77,78]. Several studies aimed at acquiring a better understanding of the biosynthesis of aflatoxins have been performed on *Aspergillus parasiticus* and *Aspergillus flavus*, which are most commonly linked to the contamination of agricultural crops [48]. The biosynthesis of aflatoxins is complex, with many regulation layers, and influenced by several environmental conditions, including humidity and temperature [48].

### 2.2. Ochratoxins

Ochratoxins are secondary metabolites of fungi produced by the *Aspergillus* and *Penicillium* genera. In their chemical composition, they contain a phenylalanine moiety and an isocoumarin moiety joined by an amide bond [79,80]. *Penicillium* is more significant in regions with a temperate climate, while the species of *Aspergillus* are more significant in the tropics and subtropics. Ochratoxin A (OTA), ochratoxin B (OTB), and ochratoxin C (OTC) are the major ochratoxins found in nature [81]. Ochratoxin A was first reported in 1965 [82]. OTA is produced by *Aspergillus ochraceus*, *A. niger*, *A. carbonarius*, and *Penicillium verrucosum*, and is found in several agricultural crops and food products, such as grains (e.g., cereals, legumes), baby foods, infant formula, coffee, milk, meat, spices, licorice, beer, wine, fruits, and nuts [3,83]. OTA is chlorinated, which is one of its distinctive characteristics [81]. OTB, which is not chlorinated, and OTC, the OTA ethyl ester, have less toxicity and are less common [80]. Production of ochratoxins by *A. ochraceus* is optimum at pH 3–10, a temperature of 31 °C, and a minimum of 0.8 water activity. Production of ochratoxins by *P. verrucosum* is optimal at pH 6–7, a temperature of 20 °C, and a minimum of 0.86 water activity [84]. Ochratoxin production is optimal in the presence of iron, zinc, and copper [85].

Contamination of grains by ochratoxins largely depends on pre-, peri-, and post-harvest conditions. OTA is mostly concentrated in husks of grains, and removing the pericarp, or outer layer, from grains is known to reduce the concentration of OTA by at least 50% [85]. Study analyzed samples of barley, hard red spring wheat, and durum wheat stored for different durations by several commercial companies involved in grain processing in the northern and northwestern US Great Plains region had OTA in about 12% of samples, and about 81% of the samples were stored for at least 6 months. In Germany, a study analyzed samples of grains between 1991 and 1993, stored for different durations, and reported that about 54% of the samples had OTA, and in 2% levels of OTA were above 3 ng/g [86]. Many studies done in Europe reported higher levels of OTA in organic crops in comparison with conventional beer, oat bran, and wheat samples meant for consumption by humans [87,88]. In countries in Europe, the average levels of contamination in foods seem low, and, so far, the highest level of OTA contamination reported was 80 mg/kg in moldy bread meant for animal feeds [81]. OTA has been detected in acha, raisins, wines, spices, cacao, legumes, rice, maize, rye, wheat, sorghum, guinea corn, and barley as well as pork products and cow milk [80]. The mold strains that produce ochratoxin A vary among crops and geographical locations [80]. Like many mycotoxins, OTA possesses high stability and is not degraded by conventional procedures of food preparation. However, exposure of contaminated samples to temperatures above 250 °C for many minutes can reduce OTA concentrations [79]. Reducing OTA concentrations to safe levels is critical but may not be achieved by common food preparation procedures.

OTA accumulates in organs of animals due to its high protein affinity, especially to albumin, and may cause contamination carryover [81]. Most individuals have detectable levels of OTA in their blood, although at extremely low levels. In Sweden and Norway, OTA was reported in samples of breast milk and plasma, while in Brazil, most breast milk samples proved OTA negative. Nearly 22% of individuals from a study in France had OTA levels range between 0.1 and 1.30 ng/mL in blood, while 97% of individuals from Italy had OTA levels range between 0.12 and 2.84 ng/mL in blood, with levels in males significantly higher compared to females [89]. In general, levels of OTA in the blood are higher in patients suffering from nephropathy compared to the healthy ones [80]. Based on studies involving animal models, OTA has been recognized as a likely human carcinogen (group 2B), and the cancer vulnerability is both species and sex specific [90].

Animal studies have shown that OTA is immunotoxic, teratogenic, neurotoxic, hepatotoxic, and nephrotoxic. OTA-acute toxicity affects the kidneys most and pigs showed the highest sensitivity with nephropathy after exposure [84]. Teratogenic effects were reported in many animal studies, including chick, quail, rabbit, hamster, rat, and mouse studies, with craniofacial abnormalities and reduced birth weight being the most common [81,90]. Days 5–7 of gestation in rats seemed extremely sensitive; a single subcutaneous OTA dose of 1.75 mg/kg within these days resulted in the maximum resorption number, the most reduction in the weight of fetuses, skeletal malformations, and the greatest amounts of soft tissue [91]. Chronic exposure to OTA in low doses may have higher toxicity than acute exposures in high doses [90].

In humans, OTA has been associated with urothelial tumors, chronic interstitial nephropathy, and Balkan endemic nephropathy (BEN). In addition, epidemiological studies have revealed early-life OTA exposure to be associated with testicular cancer [80]. Renal tumors in humans are believed to ensue when foods containing OTA levels above 70 μg/kg are consumed daily [84]. A few studies reported the epidemiological associations of ochratoxin A in feeds/foods (or its concentration in the blood) with Balkan endemic nephropathy incidence [84,89]. BEN has been reported in geographical areas like Bosnia and Herzegovina, Macedonia, Serbia, Croatia, Romania, and Bulgaria. A study from northwestern Bulgaria reported that consuming 1.21 μg of OTA per day is associated with BEN but underlined that OTA can cause this condition when in synergy with other toxins in the environment and/or during interaction with some genotypes that can predispose it [89]. Acute renal failures have been associated with inhaling ochratoxin released by *A. ochraceus* 24 h after a woman stayed 8 h with her husband (a farmer) in grain storage facility closed for many months.

In humans, the half-life of OTA can be as long as 35 days (840 h) after a single dose oral ingestion. This is believed to be described with reabsorptions in enterohepatic circulations, extensive binding to protein, or reabsorptions from urine following tubular secretions [84,85]. In animals, the half-life of OTA could be shorter, for instance in mice it can be 12 h, in pigs 48 h, and rats 150 h, except in macaques, in which it has about a 1400 h half-life [85]. OTA has shown testicular toxicities in animals and an increase in testicular cancer incidence has been shown in many regions with identified contamination of food [90]. In a study involving mice, the OTA intraperitoneal administration at 7.5 days of gestation resulted in downregulation of *Dmrt-1*, an essential tumor suppressor gene and transcription factor for the development of mammalian testicles. Downregulation of *Dmrt-1* was linked to germ cell tumors in mice testicles, while its homolog in humans was implicated in susceptibility to germ cell tumors [90,92]. In the EU, the limit of OTA permitted in imported foods is 10.0 μg/kg for instant coffee, 5 μg/kg for roasted coffee, 2 μg/kg for grape juice, 2 μg/kg for wine, 3 μg/kg for processed cereal food products, and 5 μg/kg for unprocessed cereal grains [80].

### 2.3. Trichothecenes (Trichothecene Mycotoxins)

Trichothecenes, also called trichothecene mycotoxins, are a diverse group of more than 200 sesquiterpenoid metabolites with structural similarities and have a common core structure of tricyclic 12,13-epoxytrichothec-9-ene. They are chemically related mycotoxins and are commonly found in foods such as rice, oats, rye, barley, maize, wheat, vegetables, etc. Trichothecene mycotoxins are produced by various fungal species of *Fusarium* (such as *Fusarium crookwellense*, *F culmorum*, *F graminearum*, and *F poae*), *Myrothecium*, *Verticimonosporium*, *Trichothecium*, *Trichoderma*, *Cephalosporium*, *Stachybotrys*, and *Spicellum*. The most common trichothecene is deoxynivalenol (DON), also referred to as vomitoxin [93,94]. Others include T-2 toxin, HT-2 toxin, 3- and 15-acetyldeoxynivalenol, diacetoxyscirpenol, etc. The most significant structural features responsible for the trichothecenes biological activities include the 12,13-epoxy ring, the acetyl or hydroxyl groups present at suitable positions on the nucleus of trichothecene, and the side-chain position and structure. Due to the presence of an ester–ether connection between C-4 and C-15 or of a macrocyclic ester, trichothecene mycotoxins can be nonmacrocyclic and macrocyclic. Macrocyclic trichothecenes are placed under two classes as type A and type B. The type A trichothecenes contain an ester- or hydrogen-type side chain at the position of C-8, with the inclusion of DAS (anguidine), T-2, and HT-2 (deacetylated metabolite of T-2). Members of type A have high toxicity; in mammals, T-2 is approximately 10 times more toxic than deoxynivalenol. The type B trichothecene mycotoxins contain a ketone group at the same position, e.g., nivalenol and deoxynivalenol [25,95]. Type C trichothecene mycotoxins include crotocin, while type D trichothecenes include macrocyclics [96].

Trichothecene mycotoxins are the major group among the three major types of mycotoxins (zearalenone, fumonisins, trichothecenes) produced by species of *Fusarium* [16]. They are among the mycotoxins with the most chemical diversity. Trichothecenes are amphipathic (they have both hydrophobic and hydrophilic groups), with low molecular weight (between 200 and 500 Da), and thus are absorbed easily via the skin and GI tract. They can diffuse into cells and block translation by interacting with eukaryotic ribosomes; this is their primary action mechanism [94,97]. Trichothecene mycotoxins have several action mechanisms, including inhibiting DNA, RNA, and protein synthesis as well as lipid peroxidation, apoptosis, inhibiting mitochondrial functions, neurotransmitter changes, and cytokine activation [96,98].

Exposure to trichothecenes affects nearly all key systems in vertebrates [25]. A single dose of T-2 was administered to rats and the profiling of the metabolomics showed that moderate and low doses of 2 mg/kg and 0.5 mg/kg of body weight (bw) resulted in changes in metabolism, especially in urine, while high doses (4 mg/kg bw) resulted in additional metabolic changes in the thymus, spleen, stomach, and liver as well as disturbed many pathways of metabolism and interrupted microbiota of the gut [98]. A study that exposed mice to T-2 toxin reported significant oxidative damage, oxidative protein damage, and increased lipid peroxidation in a manner that depended on time, indicating that oxidative stress is a key mechanism underlying toxicity, in vivo, with more pronounced toxicity caused by the percutaneous route, while the subcutaneous route caused less toxicity [99]. T-2 is the most toxic among all the trichothecene mycotoxins, and its toxicity to animals depends on age, dosage, species, and the route of administration [100]. Cells that actively divide are more vulnerable to T-2 toxin, which explains why the immune system and the GI tract are among the main organs targeted by T-2 [98]. Chronic and acute toxicity in rats resulted in a change in distribution of serotonin, tryptophan, and tyrosine in their brain [101]. The symptoms of chronic and acute toxicity in humans and animals include carcinogenesis, immune depression, neurotransmitter imbalances, weight loss, growth retardation, oral lesions, diarrhea, and vomiting [98,100,102]. DON effects on the immune functions include immune stimulation and immune suppression, which largely depends on concentration and the exposure duration [16]. The main in vivo and in vitro T-2 metabolite is HT-2 toxin, which is a deacetylated metabolite with similar toxicities and can be produced by deacetylation reactions carried out by many microorganisms in the intestine [16].

Alimentary toxic aleukia (ATA) in humans, which is linked to exposure to trichothecenes, was first reported in eastern Siberia in 1913 and appeared again in 1932 in many western Siberia districts. ATA as an illness is presented with fever, agranulocytosis, necrotic angina, gum bleeding, mouth bleeding, nose bleeding, diarrhea, vomiting, and abdominal pain, and is associated with a high rate of mortality. An outbreak with similar symptoms was recorded earlier in New Hampshire in the 1730s [95,97,103]. At first, the outbreak was wrongly considered an epidemic, but the idea was rejected due to the fact that none among the health practitioners that treated the diseased patients fell ill. Hypothetic analyses that suggested the cause was due to deficiencies of vitamins were also rejected [103]. Overwintering and delayed harvesting of grains were believed to have encouraged the mold growth and the mycotoxin production that led to the outbreak.

### 2.4. Deoxynivalenol (a Trichothecene)

Deoxynivalenol is the mycotoxin with the most economic importance, but not the most toxic of all mycotoxins. Corn, barley, oat, and wheat are the grains mostly affected by deoxynivalenol. Many studies done in the field showed that *Fusarium* head blight intensity has a linear relationship with DON accumulation [104]. Factors such as relative humidity, moisture, and temperature that affect the *Fusarium* head blight development also have effects on its accumulation [104]. Storing at below 14% moisture content and controlling insects are among the main strategies to avoid DON production [105]. DON can be found in foods obtained from animals, including eggs, milk, liver, and kidney. Few studies, not all, showed that deoxynivalenol can transfer from a dairy cow to its milk. Intoxication with DON results in fever, dizziness, headaches, diarrhea, vomiting, nausea, and abdominal pain [106]. In grains contaminated with *Fusarium*, the DON levels increase as the number of damaged grains increase. A study mixed healthy kernels and *Fusarium*-damaged kernels in 5% additions from 0 to 100% within 2 consecutive years and showed that after flours obtained from grains of each blend were evaluated, DON concentration increased as the number of *Fusarium*-infested kernels increased [104].

Among all the livestock species, swine are most susceptible to the toxicity of DON; other species, including dogs and cats, are affected too, and sensitivity to DON can vary with gender and age [107,108]. DON remains stable between 170 to 350 °C; no decrease in concentration was reported at 170 °C after 30 min [106]. Due to its solubility in water, levels of DON reduce during cooking of contaminated noodles/pasta as it leaches into water used for cooking but not when contaminated foods are fried in oil [106]. Animal studies have shown that prolonged exposure to DON in low doses may result in impairment of growth in children [3]. There is substantial interest to better understand the relationship between Kashin–Beck disease and trichothecenes. Kashin–Beck disease manifests as chronic degenerative osteoarthritis and affects 2.5 million people in roughly 15 provinces in southwestern and northeastern China, where it is endemic [3]. Its etiology seems multifactorial, and evidence from epidemiological studies points to deficiency of selenium and T-2 contamination of grains as playing potential key roles [109]. In vitro, T-2 promotes articular cartilage proteoglycan degradation, induces the degradation of cartilage matrices, induces the upregulation of matrix metalloproteinases, and causes chondrocyte apoptosis. Rats’ exposure to a diet low in nutrients together with T-2 toxin resulted in histological and radiographic changes similar to the lesions reported in Kashin–Beck disease patients; in rodents, the toxin resulted in degenerative articular changes [110,111,112].

### 2.5. Fumonisins

Fumonisins are a carcinogenic and toxic mycotoxin family and are structurally similar to sphinganine, a sphingolipids precursor [79,113]. Fumonisins are diesters of long-chain polyhydroxyamines and propanotricarboxylic acid [114]. Fumonisins have a long hydroxylated chain of hydrocarbon and tricarballylic acid, amino, and methyl groups; the amino group is vital for their biological activities [115]. In 1988, Fumonisins were discovered in South Africa, where they were first isolated from *Fusarium moniliforme* (currently known as *Fusarium verticillioides*) cultures, which frequently contaminates maize in all regions. Fumonisins are produced by no less than 14 other species of *Fusarium*, including *Fusarium nyagamai*, *F. oxysporum*, *F. globosum*, *F. fujikuroi*, and *F. proliferatum* [19,113,114]. The biosynthetic gene cluster of fumonisins has also been reported in *Aspergillus awamori* and *A. niger*, which mostly produce fumonisin B2 (FB2) [114]. Fumonisins are classified into four major groups, A, B, C, and P [15,116]. At least 15 fumonisins are currently known, with the most abundant and toxic of them all being fumonisin B1 (FB1) [117]. Fumonisin B3 (FB3) is also common. Only FB1, FB2, and FB3 are found in foods that are naturally contaminated [118]. While fumonisin B3 is 5-deoxy fumonisin B1, fumonisin B2 is 10-deoxy fumonisin B1 [118]. *Fusarium moniliforme* and *F. verticillioides* mostly produce fumonisin B1, although they also produce FB3 and FB2 in very low quantities [114]. Along with corn and corn food products, FB1 has been reported in asparagus, sorghum, beer, rice, soybeans, and beans [116]. Those with celiac disease have a higher risk of fumonisin exposure due to their diet based on rice and corn [114].

Fumonisins are the mycotoxins of most significance in maize, especially in wet and warm regions; levels of contamination could vary yearly [79,114]. They maintain stability at high temperatures and their levels reduce only above 150 °C. Fumonisins are produced in maize during the growth of fungi in the seeds or plant, either pre-harvest or in the initial stages of storage/drying [79]. The highest FB1 levels in dry milling of corn occur in bran fractions, followed by germ fractions, used as animal feed, and the fractions for the production of foods have the lowest levels [119]. Fumonisins B1 and B2 are least stable at pH 4, followed by pH 10 and 7, and at 175 °C after 60 min there was 90% decomposition regardless of pH [119]. Frying tortilla chips at 190 °C for 15 min led to a 67% reduction in fumonisin level, and corn muffin baking led to a 16% reduction in fumonisin levels at 175 °C, and at 200 °C there was a 28% reduction. At these temperature levels, the reduction was lesser at the core than at the surface of the muffins [119]. The production of fumonisins is optimum at a water activity (a_w_) of 0.95 to 0.99 and within temperatures of 20 °C to 30 °C [114]. The a_w_ has an essential role in the production of fumonisins by *Fusarium moniliforme* during the fungal growth on maize. At 0.85 to 0.86 a_w_, the fungus indicated nearly no quantifiable metabolic activity with no production of fumonisins. Slight changes in a_w_ exert huge effects on the production of fumonisins. A 5% decrease in a_w_ from 1.0–0.95 had no change in effect on the rate of fungal growth and led to a threefold reduction in fumonisin levels, but a 10% decrease in a_w_ from 1.0–0.90 led to a 20-fold reduction in the growth of fungi and a 300-fold reduction in the production of fumonisins [120].

Regulating the production of fumonisins may differ in different strains of fungi. *Aspergillus niger* thrives more at lower a_w_, with production of mycotoxins going up to a peak within 0.985 to 0.97, and 25 to 30 °C temperatures, while the species of *Fusarium* prefer a_w_ exceeding 0.99 and 20 to 25 °C temperatures [121]. Fumonisins are linked to atherosclerosis in monkeys, equine leukoencephalomalacia in horses, porcine pulmonary edema and pulmonary artery hypertrophy in swine, and kidney and liver cancer in rodents [113,114,117]. A porcine pulmonary edema outbreak occurred in the US when pigs were fed corn contaminated with *Fusarium verticillioides* from a crop in 1989 after anomalous conditions of climate in the Midwest led to high levels of fumonisins [15]. In swine, pulmonary edema seemed to be the result of acute left-sided heart failure mediated by perturbation in the biosynthesis of sphingolipid. This has been hypothetically suggested to be caused by the Golgi apparatus and the endoplasmic reticulum disruptions as well as the L-type calcium channel inhibition in cardiac myocytes, which reduces cardiac contractility and blocks the release of Ca^2+^ induced by Ca^2+^ [15]. Fumonisins inhibit the synthesis of sphingolipids, which are significant regulatory and structural molecules in eukaryotes [115]. The inhibitory effects can readily manifest hours after FB1 oral ingestion [118]. The effects can be described by their ceramide synthase inhibition ability (ceramide synthase controls sphingosine recycling and acylates sphinganine). This effect poses two consequences: complex sphingolipid synthesis inhibition and increased intracellular levels of sphinganine, which is usually present at low levels in cells, leading to cytotoxic effects [79,115].

Fumonisin exposure in humans can cause esophageal and liver cancer [117]. Its association with esophageal cancer was described when there was detection of fumonisins in maize grown at a home in an area with high incidence in Transkei, South Africa [113,122]. Subsequently, increased esophageal cancer risk was reported in people in China, Iran, the southeastern United States, northern Italy, and south-central Africa, where maize and maize products are commonly consumed; exposures were linked to cancer of the liver in some areas in China [114,118]. Animal studies showed that neural tube defects can be caused by fumonisin exposure. This, in addition to their capability to affect functions of folate-binding proteins and other proteins of membranes, and increased neural tube defect rates in humans in many regions with suspected or known exposure to fumonisins, increased the likelihood neural tube defects may be caused by that fumonisin exposure, although the underlying causal mechanism has not been fully established [113,117]. In southern Texas, a study done at the border between Texas and Mexico evaluated fumonisin exposure from tortillas made at home through sphinganine measuring via the ratio of sphingosine in maternal serum. A dose–response association was reported between sphinganine (sphingosine ratios of 0.11 to 0.35) and the adjusted odds neural tube defects ratio; although it was not reported for those who had the highest exposures (sphinganine: levels of sphingosine >0.35), and this group also had the least number of participants. The findings suggested a likely dose–response association between neural tube defects and maternal fumonisin exposure [113,123].

One of the crucial considerations is that fumonisins carry over from foods to maternal breast milk, followed by subsequent infants’ exposures. A study was done in northern Tanzania and reported that about 44% of samples of breast milk obtained from breastfeeding mothers contained fumonisin B1 and about 10% of them had levels that exceed the limit of 200 μg/kg set by the European Union for infant foods [124]. Along with its capability to disturb the metabolism of sphingolipids, fumonisin B1 has been reported to inhibit the mitochondrial electron transport chain complex I and encourage reactive oxygen species (ROS) generation, lipid peroxidation, and oxidative stress [117]. Additionally, fumonisin B1 was reported to have an inhibitory effect on argininosuccinate synthetase, a urea cycle enzyme that is responsible for catalyzing argininosuccinic acid formation from aspartate and citrulline [125].

The Joint FAO/WHO Expert Committee on Food Additives put the maximum tolerable fumonisin intake per day at 2 μg/kg bw for fumonisins B1, B2, and B3, in combination or alone [113]. The International Agency for Research on Cancer (IARC) has classified fumonisin B1 as possibly carcinogenic to humans (group 2B) [117]. The EU has put the maximum total fumonisins (fumonisins B1 and B2) limit at 1000 μg/kg for maize and maize products meant for direct consumption by humans, and at 800 μg/kg for snacks and breakfast cereals produced from maize. The US FDA set the total limit of fumonisins at 2 to 4 mg/kg in corn and corn products intended for human consumption, respectively, and at 3 mg/kg in corn used for popcorn [116].

### 2.6. Emerging Fusarium Mycotoxins (Enniatins, NX-2 Toxin, Beauvericin, Moniliformin, Fusaproliferin)

The recent emerging mycotoxins have become a major challenge because of their prevalent occurrence in foods such as grains, especially cereals and cereal products [126]. Emerging mycotoxins have been defined as “mycotoxins, which are neither routinely determined, nor legislatively regulated; however, the evidence of their incidence is rapidly increasing” [127]. An opinion on beauvericin (BEA) and enniatin (ENN) presence in foods and feeds was presented by the European Food Safety Authority (EFSA) with no assessment of risk because relevant toxicity data was lacking [128]. Fusaproliferin (FU), a bicyclic sesterterpene mycotoxin, is produced by species of *Fusarium*, including *Fusarium verticillioides*, *Fusarium subglutinans*, and *Fusarium proliferatum* [129]. Fusaproliferin exhibited toxicity on brine shrimp larvae and chicken embryos [129]. In terms of structure, moniliformin (MON) is a 1-hydroxycyclobut-1-ene-3,4 dione, water soluble, a small molecule, and can be produced by *Fusarium acuminatum*, *F. avenaceum*, *F. arthrosporiodes*, *F. verticillioides*, *F. chlamydosporum*, *F. redolens*, *F. oxysporum*, *F. beomiforme*, *F. thapsinum*, *F. subglutinans*, *F. sacchari*, *F. pseudoanthophilum*, *F. proliferatum*, *F. nygamai*, *F. napiforme*, *F. fujikuroi*, *F. diaminii*, *F. concentricum*, *F. bulbicola*, *F. begoniae*, *F. anthophilum*, *F. acutatum*, *F. tricinctum*, *F. ramigenum*, *F. pseudonygamai*, *F. pseudocircinatum*, *F. phyllophilum*, *F. nisikadoi*, *F. lactis*, and *F. denticulatum*, and was recently shown as one of the metabolites of *Penicillium melanoconidium* [129,130].

In terms of structure, beauvericin is a cyclic hexadepsipeptide with an alternating sequence of three N-methyl-l- and d-A-hydroxy -*iso*-valeryl-phenylalanyl residues [131]. Beauvericin was isolated from a fungus called *Beauveria bassiana* for the first time; *Beauveria bassiana* is known to cause disease in insects [131], and commonly occurs in corn and corn products infected by species of *Fusarium*. BEA infects cereals and cereal products not just in countries in Europe including the Czech Republic, Italy, Spain, and Romania, but also worldwide, including in Morocco, Iran, Rwanda, Tanzania, and Japan [132,133,134,135]. BEA has insecticidal, antifungal, and antibacterial properties, and can have toxic effects, including apoptosis induction, increased cytoplasmic calcium concentration, and fragmentation of DNA in cell lines of mammals [131].

NX-2 toxin, a new trichothecene, was recently found in cultures of rice. In terms of toxicity and structure, NX-2 is similar to 3-ADON, although it has no keto group at C-8, and as a result, NX-2 is a type A trichothecene mycotoxin [136]. The *Fusarium* species shown to produce ENNs include *Fusarium venenatum*, *F. tricinctum*, *F. torulosum*, *F. sporotrichioides*, *F. scirpi*, *F. sambucinum*, *F. poae*, *F. oxysporum*, *F. lateritium*, *F. langsethiae*, *F. kyushuense*, *F. equiseti*, *F. culmorum*, *F. compactum*, *F. merismoides*, *F. acuminatum*, *F. arthrosporioides*, and *F. avenaceum* [137]. The species of *Fusarium* with the capacity to produce enniatins occur in various geographical regions. However, the ENNs do not contaminate cereals alone, but also contaminate several foods such as coffee, tree nuts, dried fruits, beans, and vegetable oil. The most commonly detected ENNs in foods and feeds include enniatin A (ENA), enniatin B (ENB), enniatin A1 (ENA1), and enniatin B1 (ENB1) [138]. There is little or no indication that enniatins pose a concern to humans and animals; although, ENNs may have role to play in making other *Fusarium* toxins’ impact more pronounced (particularly DON) through cellular export inhibition [139]. As a result of their high prevalence in foods and feeds and their potential toxicity to humans and animals, the interest in emerging mycotoxins is increasing [131]. Studies are required for better understanding of these emerging mycotoxins, including their possible toxicities to humans and animals, as well as how to effectively reduce their presence in foods and feeds to safe levels.

### 2.7. Sterigmatocystin

Sterigmatocystin (STC), a secondary metabolite of fungi, is produced by various species of *Aspergillus*, such as *A. versicolor* (which is the major STC producer), *A. sydowi*, *A. quadrilineatus*, *A. aureolatus*, *A. amstelodami*, *A. ruber*, and *A. chevalieri*. Other mold species can also produce STC, including some from the *Penicillium*, *Emiricella*, *Chaetomium*, and *Bipolaris* genera [140]. *A. versicolor* has optimal growth at 0.95 a_w_, although it can grow below 0.8 a_w_. The mold grows between 4 °C and 40 °C, but its optimum temperature for growth is 30 °C [140]. Sterigmatocystin is a late metabolic compound in the pathway of AFB1 and, similarly to AFB1, STC contains xanthones and furan rings [15,141]. In 2007 and 2006, a study evaluated samples drawn from various grains from Latvia and reported that about 14% of samples from 2006 were positive for sterigmatocystin at levels between <0.7 and 83 μg/kg, while 35% of samples from 2007 tested positive at levels between <1 and 47 μg/kg [142]. STC occurs in moldy peanuts, corn, barley, rice, and wheat [140]. When various breads were inoculated with spores of *Aspergillus versicolor*, levels of STC around 400 μg/kg were attained in 10 days [143]. Along with human foods and animal feeds, sterigmatocystin can be detected in interior environments, including building materials from wallpaper damaged by water and carpet dusts from damp indoor environments [144].

Sterigmatocystin has teratogenic, mutagenic, and carcinogenic effects, but less potent than AFB1 and can cause hepatic toxicity in most animals. The carcinogenicity of STC is organ-specific, depending on the route of administration. Rats’ exposure to STC resulted in hepatocellular carcinoma from oral administration or intraperitoneal administration, and when applied to skin resulted in squamous cell carcinomas [25,140]. Short-term administration of STC in mice had effects on the immune functions by changing the number of plasmacytoid dendritic cells and T_reg_ [145]. A study fed dairy cattle with STC-contaminated food (8 mg/kg of STC produced by *Aspergillus versicolor*) and reported that STC induced bloody diarrhea and also caused death [140]. STC effects on humans have not been fully understood. The IARC placed STC under class 2B carcinogens [144].

### 2.8. Ergot Alkaloids

Ergot alkaloids are comprised of a complex family of the derivatives of indole produced by the Clavicipitaceae (such as *Neotyphodium* and *Claviceps*) and Trichocomaceae (such as *Penicillium* and *Aspergillus*) families [146,147]. A tetracyclic ergoline ring is their common structural characteristic. Ergot alkaloids are both harmful and beneficial to humans [147]. Ergot alkaloids, both natural and semisynthetic, are used in several medicines [146]. There are many cases of widespread ergot alkaloid poisoning; ergot alkaloids were reported to be responsible for the Massachusetts Salem Witch Trials [147]. In 1692, after many female teenagers were affected by delirious seizures and fits, traditional physicians blamed the cause on witchcraft. Innocent individuals were grossly accused of practicing witchcraft, tried, convicted, and then executed; however, records were later evaluated and showed that ergot alkaloids produced by *Claviceps purpurea* may have caused the intoxication [148]. Ergot alkaloids have also been implicated in several witchcraft accusations and trials [147,148,149].

**Figure 2:**
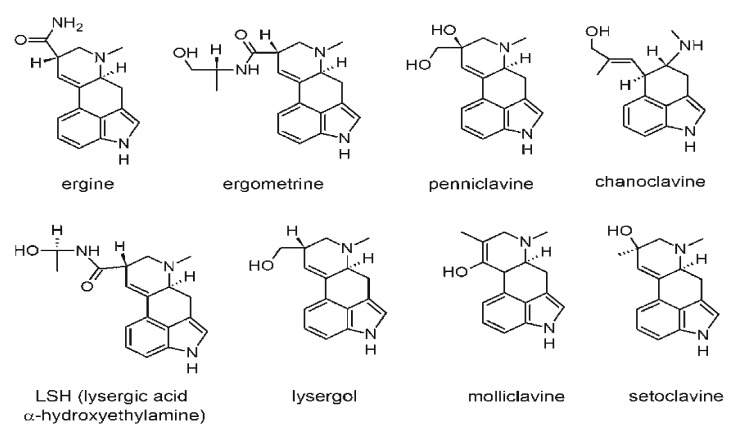
Chemical structures of some common ergot alkaloids (Reprinted/Adapted from sources [1,9,151,153]).

The first documented outbreak of ergotism was occurred in France from 944–945 AD after about 20,000 individuals in the Aquitaine region lost their lives due to poisoning, and many outbreaks were recorded in the 16th century in Germany. Two different kinds of toxic reactions were reported over the course of these outbreaks. The first one, also called the gangrenous form and usually known as “St. Anthony’s Fire”, commonly occurred in France and was characterized by gangrene with burning pain but no loss of blood, a marked peripheral vasoconstriction, and the swelling of feet, hands, and limbs [150]. The name “St. Anthony’s Fire” was derived from the St. Anthony monastic order, whose members administered treatment to the disease sufferers. Although the cause was not known during at the time, people noticed that treatment and pilgrimage at monasteries cured the ailment. Currently, it has been made known that the ailment was caused by ergot exposure from rye consumption, and while in pilgrimage people’s food sources changed and they were not exposed anymore [151,152]. The second reaction, called the convulsive form, was commonly reported in Germany. Patients presented with hallucinations and delirium accompanied by severe diarrhea, muscle spasms, convulsions, and rigid, very painful limbs [150]. Figure 2 shows some common ergot alkaloids [1,9,151,153].

Modern techniques used for grain cleaning have largely eliminated ergotism as a disease in humans, however, it still poses threat to many animals such as chickens, pigs, cattle, and sheep [153]. Livestock exposure to ergot alkaloids results in gangrenous extremities, agalactia, ataxia, abortion, and convulsions [152,153]. The similarity in structure between the biogenic amines and tetracyclic ergoline ring gives ergot alkaloids the property to act on the α-adrenergic, serotoninergic, and dopaminergic receptors. Through the activation of the pituitary D2 dopamine receptors, ergot alkaloids can cause vasoconstriction, along with the loss of hooves, tails, and ears [153]. Both gangrenous and convulsive types of ergotism can be attributed to the capability of ergot alkaloids to cause vasoconstriction [153].

### 2.9. Zearalenone

Zearalenone (ZEA), also called 6-(10-hydroxy-6-oxo-*trans*-l-undecenyl)-β-resorcyclic acid lactone, and formerly referred to as F-2 toxin, is produced by species of *Fusarium*, such as *Fusarium crookwellense*, *Fusarium cerealis*, *Fusarium semitectum*, *Fusarium equiseti*, *Fusarium graminearum*, and *Fusarium culmorum*, which are known to contaminate cereals worldwide [154,155]. Maize is the most contaminated cereal, although the mycotoxin has also been found in soybean, rice, rye, sorghum, oats, barley, and wheat products [156]. Structurally, zearalenone is similar to 17β-estradiol; its capacity to bind estrogen receptors competitively shows the observed alterations in the reproductive tract and its capacity to lead to fertility impairment in guinea pigs, rabbits, hamsters, rats, mice, and domestic animals [16,26,156]. In humans, zearalenone is also linked with hypoestrogenic syndromes [156]. ZEA is mostly formed before harvesting and its synthesis can continue if the agricultural commodities are stored in poor conditions [155]. The ZEA–estrogen receptor complex is translocated to the nucleus where the complex binds to responsive elements of steroids, regulating many gene transcriptions [154]. Zearalenone or its metabolic compounds are known to bind transcription factors, including pregnane X receptors involved in expressing enzymes in pathways of biosynthesis [154].

In mice, guinea pigs, and rats, zearalenone showed low acute toxicity when orally administered, but showed more toxicity following intraperitoneal administration. ZEA chronic administration can cause uterine fibroids, pituitary adenomas, hepatocellular carcinoma, and liver damage in mice, and chronic progressive hematotoxicity, testicular atrophy, cataracts, retinopathy, and nephropathy in rats [156]. Studies done in vitro showed that zearalenone forms DNA adducts, and ZEA’s intraperitoneal administration in mice resulted in DNA adduct formation in the liver and kidneys [156]. Among farm animals, pigs are most sensitive to zearalenone, and some clinical consequences of ZEA exposure include stillbirth, decreased fertility, persistent corpora lutea, prolonged estrus intervals, and ovarian atrophy [154]. In male pigs, zearalenone induces feminization, decreases spermatogenesis, decreases testicular weight, decreases libido, and decreases testosterone levels [156].

A study evaluated the concentrations of ZEA in the specimens of endometrial tissue and found that women with hyperplasia had lower concentrations than women with adenocarcinoma, and the mycotoxin was not found in women with no endometrial changes, suggesting the likelihood that ZEA may be involved in carcinogenesis in humans [157]. An interesting ZEA characteristic is its antagonistic effect on other mycotoxin toxicities. OTA-induced kidney damage was significantly lessened in rats co-administered with both OTA and ZEA [26]. Zearalenone and deoxynivalenol are produced by the same species of fungi and thus they could co-contaminate foods and crops. While deoxynivalenol has proinflammatory activities, zearalenone seems to have anti-inflammatory activities through the suppression of NF-κB transcription factor activation, which probably explains most of the antagonistic effects [154].

The structures of most mycotoxins could be changed due to their metabolisms in plants where they are produced. Due to newly attained physical and chemical properties, their presence may be underreported during the analysis of samples. These are referred to as masked mycotoxins. Masked mycotoxin presence may be underestimated due to modifications in the antibodies-recognized epitope, their chromatographic behavioral changes, or polarity changes that impair their extraction with solvents [158]. The abundant presence of α-zearalenol (a more estrogenic ZEA derivative) is usually not estimated and unregulated by legislation, resulting in underestimating the risks of its hyperestrogenic effects [158,159].

### 2.10. Alternaria Toxins (Altenuene, Tentoxin, Tenuazonic Acid, Altertoxin, Alternariol Methyl Ether, Alternariol)

The species of *Alternaria* can be seen ubiquitously and in several ecosystems, including soil, atmosphere, agricultural commodities, seeds, and plants [160]. *Alternaria* species produce *Alternaria* toxins, which usually contaminate foods during storage, with tenuazonic acid (TeA), altertoxins (ATXs), altenuene (ALT), tentoxin (TEN), alternariol methyl ether (AME), and alternariol (AOH) being the most significant toxins [161]. Other *Alternaria* toxins include altenuisol (AS), altersetin (ALS), stemphyltoxin (STE), alteichin or alterperylenol (ALTCH), etc. Over 70 secondary metabolic compounds are produced by the *Alternaria* species that produce toxins, such as *Alternaria triticina*, *Alternaria tenuissima*, *Alternaria solani*, *Alternaria japonica*, *Alternaria dauci*, *Alternaria brassicae*, and *Alternaria alternata* [160,162]. Additionally, more than 30 mycotoxins have been isolated and belong to various classes depending on their chemical structure [161]. The *Alternaria* genus includes pathogenic, endophytic, and saprophytic species; *Alternaria* is a cosmopolitan fungus that occurs in anthropogenic and natural environments [160]. *Alternaria alternata* is the most common among the *Alternaria* species in fruit and vegetables after harvesting, and also the most significant species that produces mycotoxins [162]. While ATXs are a member of the perylene quinone derivatives, ALT, AME, and AOH are members of the dibenzo-α-pyrone derivatives [163]. TeA is a member of the tetramic acid derivatives that have phytotoxic and antibacterial properties and is responsible for cases of acute toxicity in dogs, chicken, and mice in addition to hematological disorders in humans [164].

The most commonly studied *Alternaria* toxins include TeA, AME, and AOH [162]. Although most *Alternaria* toxins show low acute toxicities, AME and AOH are mostly toxic because of their genotoxic, cytotoxic, carcinogenic, and mutagenic effects, with scientific-based findings from toxicological studies in vitro involving mammalian and bacterial cells. AOH has been shown to have more genotoxicity in carcinoma colon cells of humans than AME [164]. At present, monitoring guidelines or regulatory limits have not been fully established for *Alternaria* toxins in foods worldwide. After an EFSA study, the toxicological concern threshold (TTC approach) was put into use by the EFSA due to little or no data on *Alternaria* toxin toxicities with the aim of assessing the concern levels for humans [162]. For genotoxic *Alternaria* toxins (AME and AOH), a 2.5 ng/kg body weight per day TTC value was set, while for non-genotoxic *Alternaria* toxins (TEN and TeA), a 1500 ng/kg body weight per day TTC value was set; these estimates of exposures are not likely to pose a concern to humans [162].

**Figure 3:**
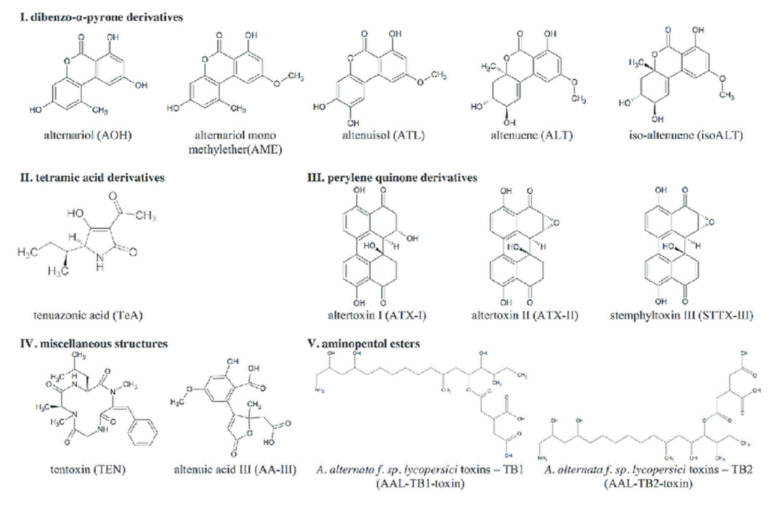
The chemical structures of common *Alternaria* toxins (Reprinted/Adapted from sources [1,7,164,165]).

The chemical structures of common *Alternaria* toxins are shown in Figure 3 [1,7,164,165]. The substrate composition, pH, a_w_, and temperature are the most significant abiotic and biotic parameters that affect mycotoxin biosynthesis and, consequently, *Alternaria* toxin biosynthesis. The pH and a_w_ in particular affect most *A. alternata* biosynthesis [164]. Studies were done using red wine, juice samples, dried and fresh tomatoes, wheat and wheat products, and dried fruits. *Alternaria* toxins of interest in most studies include ALT, TeA, TEN, AME, and AOH [163]. *Alternaria* toxin occurrence has been reported in several countries including Italy, the Netherlands, China, Canada, Argentina, and Germany [1,163,164]. *Alternaria* toxins are found in many food commodities, including beer, fruit juices, vegetable juices, wine, peppers, fresh and dried tomatoes, flour, bran, wheat, dried fruit, cereal products (e.g., rice and oat flake), sunflower oil, and sunflower seeds [163,165,166].

More scientific-based studies are still being done on *Alternaria* toxins. The species of *Alternaria* are black molds with worldwide distribution, and one-fourth of more than 120 secondary metabolites known thus far are mycotoxins [167]. Fungal species belonging to the *Alternaria* genus have been shown to have extensive distribution in plants as well as in decaying fruits and vegetables, and as their growth occurs at low temperatures, they affect refrigerated products as well [168]. *Alternaria* toxin exposure was associated with esophageal cancer in South Africa and in the Shanxi province of China [168]. At least 30 toxic metabolic compounds have been isolated from several species of *Alternaria*; most significant metabolites of *Alternaria* species include alternariol monomethyl ether, alternariol, and altenuene, which are derivatives of dibenzopyrone; altertoxins III, II, and I, which are derivatives of perylene; and tenuazonic acid, a derivative of tetramic acid [169,170]. The EFSA advised in 2012 that *Alternaria* toxins pose a serious concern to public health, while a surveying study done in the Netherlands reported that many food products contain *Alternaria* toxins [170]. A study done in Germany on wheat samples freshly harvested in winter and obtained from commercial farms between 2001 and 2010 showed that the most common of all *Alternaria* mycotoxins was tenuazonic acid [169]. Oilseeds, tomatoes, and vegetables are mostly susceptible to *Alternaria* species contamination, and *Alternaria* mycotoxins have also been found in fruit juices, olives, apples, and wheat [167,170]. In both animals and humans, *Alternaria* mycotoxin exposure has been reported to have cytotoxic, carcinogenic, mutagenic, and genotoxic properties [171].

### 2.11. Patulin (PAT)

Patulin (PAT) is a fungal metabolite and organic compound classified as a polyketide. Patulin has a heterocyclic lactone (4-hidroxi-4*H*-furo [3,2-c]piran-2(6*H*)-ona) structure and a 154.12 g/mol molecular weight as well as low volatility [172,173]. PAT can be produced by at least 60 species of fungi, including *Penicillium expansum* (*Penicillium leucopus*), *A. clavatus*, *Penicillium patulum* (*Penicillium griseofulvum* and *Penicillium urticae*), and *Penicillium crustosum*, while the most common producer of PAT is *Penicillium expansum* [172]. *Penicillium expansum* significantly influences the patulin levels produced. Mutagenicity, teratogenicity, carcinogenesis, immunotoxicity, and neurotoxicity are chronic and acute effects patulin showed on cell cultures [174]. PAT causes neurotoxic and immunotoxic effects in animals, but no reliable evidence has shown its carcinogenicity to humans [172]. The US, EU, and Chinese authorities all set 50 μg/L/kg as the patulin upper limit in fruit and apple juices [175]. The European Union established a 50 μg/kg maximum level for concentrated fruit juices, including cider, spirit drinks, fruit and reconstituted nectars, and other apple-derived fermented drinks or those with apple juice. The European Union established a 25 μg/kg maximum level for solid apple products, such as apple puree and apple compote, aimed at direct consumption by adults. The European Union also established a 10 μg/kg maximum level for solid apple products and apple juice, inclusive of apple puree and apple compote, for young children and infants [176]. In 1995, the Joint FAO/WHO Expert Committee on Food Additives (JECFA) implemented a 0.4 mg/kg body weight per day provisional maximum tolerable daily intake (PMTDI) for patulin [172]. Patulin is seen in fruits and vegetables, apples and apple products in particular, in several regions worldwide, and sporadically in other fruits, including grapes, oranges, and pears as well as their products. Patulin was first evaluated as potential antibiotic, but further studies have shown it to be toxic to humans, causing hemorrhages, ulcerations, vomiting, and nausea. The United States, China, and the European Union present most significant PAT contamination problems because they remain the main apple and apple product producers [174].

**Table 1:** Major common mycotoxins, their (established/evolving) toxicities, and maximum allowable limits.

Mycotoxin	Common Fungal Species	Foods Where Commonly Found	Toxicities	Maximum Allowable Limits and Associated Remarks	Reference(s)
Aflatoxins (aflatoxins B1, B2, G1, G2, M1, M2)	*Aspergillus parasiticus*, *Aspergillus flavus*, *Aspergillus bombycis*, *A. pseudotamarii*, *A. nomius*, etc.	Cereals, legumes, fruits, seeds, vegetables, nuts, etc.	Liver cancer; hepatocellular carcinoma; target DNA; mutagenic and teratogenic effects	The EU set limits of 4 μg/kg and 2 μg/kg for total aflatoxins and AFB1 permitted, respectively, in nuts, dried fruits, and cereals meant for direct consumption by humans. The AFM1 maximum residue level in milk is set by the European Union and the United States at 50 ng/kg and 500 ng/kg of raw milk, respectively. The AFB1 maximum residue level in feeds of lactating cows is set at 5 μg AFB_1_/kg, 10 μg/kg, and 20 μg/kg of feeds in the EU, in China, and in the US, respectively	[19,26,27,34,176]
Ochratoxins (ochratoxins A, B, C)	Species of *Aspergillus* and *Penicillium*, including *Aspergillus ochraceus*, *Aspergillus niger*, *Aspergillus carbonarius*, *Penicillium verrucosum*	Cereals, legumes, seeds, fruits, vegetables, nuts, etc.	Immunotoxic, teratogenic, neurotoxic, hepatotoxic, and nephrotoxic activities; nephropathy in pigs; in humans, ochratoxin A was linked to urothelial tumors, chronic interstitial nephropathy, renal failure, and Balkan endemic nephropathy; etc.	In the EU, OTA limits in imported foods are set to a maximum of 10.0 μg/kg for instant coffee, 5 μg/kg for roasted coffee, 2 μg/kg for grape juice, 2 μg/kg for wine, 3 μg/kg for processed cereal food products, and 5 μg/kg for unprocessed cereal grains	[1,81]
Trichothecenes (trichothecene mycotoxins), examples include deoxynivalenol (vomitoxin), 3- and 15-acetyldeoxynivalenol, nivalenol, anguidine, T-2 toxin, HT-2 toxin, crotocin, diacetoxyscirpenol, macrocyclics, etc.	Species of *Fusarium* (*Fusarium crookwellense*, *F culmorum*, *F graminearum*, *F poae*), *Myrothecium*, *Verticimonosporium*, *Trichothecium*, *Trichoderma*, *Cephalosporium*, *Stachybotrys*, and *Spicellum*	Rice, oats, rye, barley, maize, wheat, vegetables, etc., and animal foods, including eggs, milk, liver, and kidneys	They can diffuse into cells and block translation by interacting with eukaryotic ribosomes; this is their primary action mechanism. Other action mechanisms for toxicity include inhibiting DNA, RNA, and protein synthesis, lipid peroxidation, apoptosis, inhibiting mitochondrial functions, neurotransmitters changes, and cytokine activation. Exposure to trichothecenes affects nearly all key systems in vertebrates … alimentary toxic aleukia (ATA) in humans, etc.	The US FDA has established a level of 1 ppm restriction for deoxynivalenol. The range of TDI of 100 ng/kg bw for the sum of T-2 and HT-2 toxins is used by the EFSA.	[16,96,98]
Fumonisins (fumonisins B1, B2, B3, etc.)	*Fusarium* species such as *Fusarium verticillioides*, *Fusarium nyagamai*, *F. oxysporum*, *F. globosum*, *F. fujikuroi*, *F. proliferatum*, *Aspergillus awamori*, *A. niger* etc.	Along with corn and corn food products, fumonisins have been reported in asparagus, sorghum, beer, rice, soybeans, beans, etc.	Fumonisins are linked to atherosclerosis in monkeys, esophageal and liver cancer in human, equine leukoencephalomalacia in horses, porcine pulmonary edema and pulmonary artery hypertrophy in swine, and kidney and liver cancer in rodents. Fumonisins inhibit sphingolipids synthesis.	The International Agency for Research on Cancer (IARC) has classified fumonisin B1 as possibly carcinogenic to humans (group 2B). The EU has put the maximum total fumonisin (fumonisins B1 and B2) limit at 1000 μg/kg for maize and maize products meant for direct consumption by humans and at 800 μg/kg for snacks and breakfast cereals produced from maize. The US FDA set a total limit of fumonisins at 2 to 4 mg/kg in corn and corn products intended for human consumption and at 3 mg/kg in corn used for popcorn. The Joint FAO/WHO Expert Committee on Food Additives put the maximum fumonisins tolerable intake per day at 2 μg/kg bw for fumonisins B1, B2, and B3, in combination or alone.	[19,114,116,117]
Emerging *Fusarium* mycotoxins (enniatins, NX-2 toxin, beauvericin, moniliformin, fusaproliferin, etc.)	Species of *Fusarium*, including *Fusarium verticillioides*, *Fusarium subglutinans*, *Fusarium proliferatum*, *Fusarium acuminatum*, *F. avenaceum*, *F. arthrosporiodes*, *F. chlamydosporum*, *F. redolens*, *F. oxysporum*, *F. beomiforme*, etc.; *Beauveria bassiana*	Corn, rice, corn products, seeds, nuts, coffee, tree nuts, dried fruits, beans, vegetable oil, etc.	As a result of their high prevalence in foods and feeds and their potential toxicity to humans and animals, the interest in emerging mycotoxins is increasing. Beauvericin has insecticidal, antifungal, and antibacterial properties and can have toxic effects, including apoptosis induction, increased cytoplasmic calcium concentration, and fragmentation of DNA in cell lines of mammals.	Not available	[126,127,133]
Sterigmatocystin	*Aspergillus* species, such as *A. versicolor* (major producer), *A. sydowi*, *A. quadrilineatus*, *A. aureolatus*, *A. amstelodami*, *A. ruber*, *A. chevalieri*, as well as species of *Penicillium*, *Emiricella*, *Chaetomium*, and *Bipolaris*	Peanuts, corn, barley, rice, wheat, grain products, etc.	Sterigmatocystin has teratogenic, mutagenic, and carcinogenic effects but is less potent than AFB1 and can cause hepatic toxicity in most animals; hepatocellular carcinoma and squamous cell carcinomas in rats; bloody diarrhea and death in cattle; LD50 in mice is 800 mg/kg and above	The IARC placed STC under class 2B carcinogens. The California Department of Health Services used values TD50 from the Cancer Potency Database to produce “no significant risk” intake levels for humans. The resulting level was 8 mcg/kg bw per day for a 70 kg adult. No limit has been made available in many countries	[140,144]
Ergot alkaloids	Comprised of a complex family of the derivatives of indole produced by the Clavicipitaceae (such as *Neotyphodium* and *Claviceps*) and Trichocomaceae (such as *Penicillium* and *Aspergillus*) families. *Claviceps purpurea* is the dominant producer	Rye (most common host), triticale, barley, wheat, oats, etc.	Causes ergotism; ergot alkaloids are both harmful and beneficial to humans; can cause delirious seizures, fits, St. Anthony’s Fire, etc.; can cause gangrenous and convulsive forms of toxicities	Maximum tolerable limits are in the EU commission pipeline while current ergot sclerotia content is set in unprocessed cereals at a maximum of 0.05%. In the US, rye and wheat are considered unsafe for consumption by humans if they contain above 0.3% sclerotia by weight, and barley, triticale, or oats are graded when they contain above 0.1%. The maximum ergot level set by the European Union is 0.05% in common wheat and durum, i.e., 500 mg/kg *w*/*w* sclerotia.	[147]
Zearalenone (formerly referred to as F-2 toxin)	Species of *Fusarium*, such as *Fusarium crookwellense*, *Fusarium cerealis*, *Fusarium semitectum*, *Fusarium equiseti*, *Fusarium graminearum*, *Fusarium culmorum*, etc.	Maize, soybean, rice, rye, sorghum, oats, barley, wheat, grain products, etc.	Zearalenone or its metabolic compounds are known to bind transcription factors, including pregnane X receptors involved in expressing enzymes in pathways of biosynthesis; zearalenone chronic administration can cause uterine fibroids, pituitary adenomas, hepatocellular carcinoma, and liver damage in mice, and chronic progressive hematotoxicity, testicular atrophy, cataracts, retinopathy, and nephropathy in rats; among other animals, pigs are more prone its toxicities	The tolerable daily intake (TDI) for zearalenone was set by the EFSA at 0.25 μg/kg bw/day, and is also recommended by other international bodies such as the Joint FAO/WHO Expert Committee on Food Additives (JECFA)	[155,156]
*Alternaria* toxins (altenuene, tentoxin, tenuazonic acid, altertoxin, alternariol methyl ether, alternariol)	*Alternaria* species such as *Alternaria triticina*, *Alternaria tenuissima*, *Alternaria solani*, *Alternaria japonica*, *Alternaria dauci*, *Alternaria brassicae*, *Alternaria alternata*	Fruits and vegetables, seeds, grains, plants, beer, fruit juices, vegetable juices, wine, peppers, fresh and dried tomatoes, flour, bran, wheat, dried fruit, cereal products (e.g., rice and oat flake), sunflower oil, sunflower seeds, etc.	Tenuazonic acid has phytotoxic and antibacterial properties and acute toxicities for dogs, chicken, and mice, in addition to hematological disorders in humans. Although most *Alternaria* toxins show low acute toxicities, alternariol methyl ether and alternariol are mostly toxic because of their genotoxic, cytotoxic, carcinogenic, and mutagenic effects, with scientific-based findings from toxicological studies in vitro involving mammalian and bacterial cells.	The toxicological concern threshold (TTC approach) was put into use by the EFSA; for genotoxic *Alternaria* toxins (AME and AOH), a 2.5 ng/kg body weight per day TTC value was set, while for non-genotoxic *Alternaria* toxins (TEN and TeA), a 1500 ng/kg body weight per day TTC value was set	[162,164]
Patulin	*Penicillium expansum*, *A. clavatus*, *Penicillium patulum* (*Penicillium griseofulvum* and *Penicillium urticae*), *Penicillium crustosum*, etc.	Apples, apple products, fruits, vegetables, cereals, legumes, seeds, nuts, etc.	Mutagenicity, teratogenicity, carcinogenesis, immunotoxicity, and neurotoxicity are chronic and acute effects patulin showed on cell cultures. PAT causes neurotoxic and immunotoxic effects in animals, but no reliable evidence has shown its carcinogenicity to humans. However, studies have shown human toxicities, such as hemorrhages, ulcerations, vomiting, and nausea	The US, EU, and Chinese authorities all set 50 μg/L/kg as the patulin upper limit in fruit and apple juices. The EU established a 10 μg/kg to 50 μg/kg limit depending on the type of food	[164,174,175]
Other common mycotoxins (tremorgenic mycotoxins, fusarins (fusarins A–F), 3-nitropropionic acid, cyclochlorotine, sporidesmin)	Tremorgenic mycotoxins are produced by *Aspergillus terreus*, species of the *Penicillium* genus, etc.; *Pithomyces chartarum* produces sporidesmin; cyclochlorotine is produced by *Penicillium islandicum*; 3-nitropropionic acid (3-NPA) is produced by the species of *Arthrinium*; fusarins are produced by the species of *Fusarium*, such as *Fusarium verticillioides* (formerly *Fusarium moniliforme*), *Fusarium graminearum* (*Fusarium venenatum*), *Fusarium poae*, *Fusarium sporotrichioides*, *Fusarium oxysporum*	Several foods and feeds	Tremorgenic mycotoxins cause “staggers syndrome” in livestock and are linked to neurological conditions, such as seizures, tremors, mental confusion, and even death in humans. Fusarins are mutagenic; 3-nitropropionic acid interjects mitochondrial electron transport; Cyclochlorotine interrupts myofibrils and is hepatotoxic in animals; due to the hydrophobicity of sporidesmin, it can be integrated easily into the membranes of cells, in which it changes the organization of the bilayer	The EU, the US, the WHO, etc., all have various limits for these mycotoxins	[175,176,177,178]

### 2.12. Other Common Mycotoxins

Tremorgenic mycotoxins are group of mycotoxins that cause convulsions, ataxia, muscle tremors, and confusion, sometimes resulting in death. Tremorgenic mycotoxins are a challenge to agriculture/livestock, causing many neurological conditions generally termed “staggers syndrome”. Tremorgenic mycotoxins pose health concerns to humans, demonstrating neurological conditions like seizures, tremors, mental confusion, and even death. Clinical manifestations could either be mild, severe, or life-threatening [179,180]. Mycotoxins in this group include territrems A and B, which are produced by the fungus *Aspergillus terreus*, and roquefortine C and penitrems A and E, produced by fungal species of the *Penicillium* genus, especially *P. crustosum* [175].

Fusarins (fusarins A–F) are a class of mycotoxins with a pentane chain with a 2-pyrrolidone moiety substituted. Fusarins are produced by the species of *Fusarium*, such as *Fusarium verticillioides* (formerly *Fusarium moniliforme*), *Fusarium graminearum* (*Fusarium venenatum*), *Fusarium poae*, *Fusarium sporotrichioides*, and *Fusarium oxysporum* [176,181]. Fusarin C is among the most widely studied fusarin mycotoxins, found in animal feeds as well as foods, and it is also mutagenic after it has undergone metabolic activation [181,182,183]. A mycotoxin produced by the species of *Arthrinium,* 3-nitropropionic acid (3-NPA) interposes mitochondrial electron transport through the irreversible inhibition of succinate dehydrogenase (complex II), leading to a deficit in cellular energy [183,184,185]. In animals, 3-NPA damages the peripheral nerves, spinal tracts, hippocampus, and basal ganglia [186]. Additionally, 3-nitropropionic acid was associated with a condition called “moldy sugarcane poisoning”, which is reported to have occurred around 1972 to 1988 in China’s 13 provinces. This was believed to be due to the consumption of the already mold-infested sugarcane no less than two months post-storage. Symptoms manifested roughly 3 h post-consumption of mold-infested sugarcane as acute encephalopathy and subsequently as delayed dystonia, mostly in young adults and children. Intoxication resulted in irreversible generalized dystonia in some children. Others presented symptoms after acute intoxication, including convulsions, carpopedal spasms, dystonia, abdominal pain, diarrhea, vomiting, and nausea, resulting in a coma and even death. Intoxication in adults mostly led to problems in the gastrointestinal tract and encephalopathy was uncommon. It is, however, unknown if this variation can be attributed to the higher sugarcane level usually consumed by children or if it mirrors the differences in susceptibility/vulnerability [183,185,186].

Cyclochlorotine is a secondary fungal metabolite produced by *Penicillium islandicum*. In animals, it is hepatotoxic and in vitro studies on myoblasts have shown that cyclochlorotine would interrupt the myofibrils to form alpha-actinin aggregates and islands of myosin [187,188]. *P. islandicum* also produces another mycotoxin known as luteoskyrin, which appears regularly in rice and has been shown to elevate the serum transaminases, damage the hepatocellular membrane, and cause lipid peroxidation in mice [172,189,190]. *P. islandicum* also produces rubroskyrin, which obstructs the mitochondrial respiration in rat livers [172,189,190]. *P. islandicum* also produces rugulosin, a hepatotoxin in animals [172,189,190].

*Pithomyces chartarum* produces sporidesmin, a mycotoxin that belongs with epidithiopiperazine-2,5-dione fungal toxins [173,191]. Due to the hydrophobicity of sporidesmin, it can be integrated easily into the cell membranes, where it changes the bilayer organization [191]. Studies conducted in sheep showed the administration of sporidesmin via the oral route causes pathological changes in many organs, body weight reduction, liver toxicity, facial eczema, and photosensitization [192]. Essentially, sporidesmin could also affects cows and bring about high levels of the mycotoxin, which could appear in the remains of seemingly unaffected animals. The toxicity of sporidesmin tends to be cumulative with great variations based on individuals’ vulnerability [192]. Additionally, sporidesmin can generate hydrogen peroxide, hydroxyl radicals, and superoxide radicals [193].

## 3. Action Mechanisms of Mycotoxins: Key Aspects

Mycotoxins exhibit their cellular/molecular effects via several mechanisms. Some major types of such cellular/molecular mechanisms can include: (a) ribosomal binding, (b) protein interaction, (c) DNA effects, (d) ionophore activity, (e) metabolic enzyme inhibition, (f) rffects on hormones, (g) epigenetic properties, (h) RNA polymerase effects, (i) necrosis and apoptosis, and (j) mitochondrial interactions. These are touched briefly below:

(a) Ribosomal binding: Trichothecene toxicities occur due to their capability to bind the eukaryotic ribosomes’ 60S subunit and inhibit the reaction of peptidyl transferase [80]. Ochratoxin A competes with phenylalanine–tRNA ligase and inhibits the synthesis of protein; both aspartame and phenylalanine reduce toxicity of OTA by competing with it [95,97].
(b) Protein interaction: The plasma albumin binds to aflatoxins. After oxidation of AFB1 by cytochrome P450s, two epoxides are formed and they react with the lysine ε-amino group forming AFB1–albumin adducts [15,194,195]. Aflatoxins are immunosuppressive, and in several studies they suppressed immune response mediated by the cell and impaired phagocytosis and chemotaxis. Most immunotoxic properties of fumonisin B1 may be a result of its capability to alter the levels of mRNA and/or expression of IL-1β, IFN-γ, and TNF-α as shown in several scientific experiments [196,197]. Penitrem obstructs uptake of glutamate and GABA (γ-aminobutyric acid) into cerebellar synaptosomes, modulating the function of GABA receptors. One of the ways patulin exerts its toxicities is by causing a dose- and time-dependent phosphorylation increase of c-Jun N-terminal kinase, protein kinases 1 and 2 regulated via extracellular signal, and p38 kinase, contributing to downstream effects, including cell death and DNA damage [197,198]. A mycotoxin known as secalonic acid D, which causes “cleft palate”, phosphorylates the binding protein of the cAMP response element [198,199].
(c) DNA effects: There are two major types of interactions between nucleic acids and mycotoxins; reversible and noncovalent or irreversible and covalent [29,200]. The covalent and irreversible interaction between DNA and AFB1 results in the formation of N^7^-guanine adducts [29,200].
(d) Ionophore activity: Beauvericin and enniatins that are produced by species of *Fusarium* have ionophoric activities specific to potassium and cause an influx of potassium into the matrix of the mitochondria followed by swelling of the mitochondria [201].
(e) Metabolic enzyme inhibition: OTA, citroviridin, and AFB1 affect the metabolism of carbohydrates, while rubratoxin B and trichothecenes interfere with the metabolism of lipids [117,125]. The checmical structure of fumonisins has a high similarity to those of sphinganine and sphingosine, the sphingolipid backbones. Consequently, fumonisins inhibit ceramide synthase competitively. Fumonisin B1 inhibits argininosuccinate synthetase [125].
(f) Effects on hormones: ZEA has a structural similarity to 17β-estradiol; the effects of ZEA on receptors of estrogen can explain fertility problems in humans and animals [26,202]. Ergovaline, an ergot alkaloid, reduces levels of prolactin in animals by acting as an agonist of dopamine [202].
(g) Epigenetic properties: Few mycotoxins can change the levels of DNA methylation [203]. In mice fed for 4 weeks using maize contaminated with mycotoxin (ZEA, aflatoxin, and DON), the oocyte histone methylation and DNA methylation changed [203].
(h) RNA polymerase effects: AFB1 has inhibitory effects on chromatin-bound RNA polymerase, which is DNA-dependent, and consequently interferes with synthesis of RNA [204,205]. Luteoskyrin and patulin also inhibit RNA polymerase [204,205].
(i) Necrosis and apoptosis: AFB1 cytotoxic effects in lymphocytes of humans involve necrosis, caspase activation, and apoptosis [206], which lead to programmed cell death and irreversible cell damage. The death of cells induced by necrosis does not follow the signal transduction pathway of apoptosis.
(j) Mitochondrial interactions: Fumonisin B1 was found to obstruct the mitochondrial complex I in human neuroblastoma cells and rat primary astrocytes, resulting in reduced cellular and mitochondrial respiration and an increase in reactive oxygen species (ROS) generation with calcium signaling deregulation [207,208]. By binding covalently to the enzyme active site, 33-NPA permanently deactivates succinate dehydrogenase. Acrebol, from *Acremonium exuviarum*, inhibits mitochondrial complex III, consequently causing ATP depletion by inhibiting the chain of respiration [207,209].

## 4. Mycotoxin Prevention Measures, Decontamination, and Detoxification Approaches

Tackling mycotoxin contamination of agricultural commodities remains among the key challenges that confront many countries across the globe, which has led to various preventive measures, according to Afsah-Hejri, Hajeb, and Ehsani [210]. Further, these researchers reviewed how pre-and post-harvest tactics aimed to achieve mycotoxin degradation/detoxification. Given that these researchers discussed the physical, biological, and chemical detoxification methods of degrading mycotoxins via ozone, there is a need to look at it in a broader context [211]. Nonetheless, toxic substances in plant- and animal-based foods, along with respective components, would continue to be influenced by pre-and post-harvest processes [212,213,214,215]. Notably, pre-harvest strategies aim to avoid the development of toxigenic fungi and, hence, mycotoxins. However, once mycotoxins are produced, detoxification of foods should be based on post-harvest practices [216]. In the following sub-sections, we delve more into the pre-and post-harvest preventive measures using a broader perspective.

### 4.1. Pre-Harvest Preventive Measures

Pre-harvest preventive measures often involve good agricultural practices (GAPs), including proper irrigation and using healthy seeds as well as applying fungicides in some cases [217]. Other key pre-harvest measures are management of fertilization/irrigation, crop rotation, using resistant varieties of crops, avoiding insect damage, prevention of overwintering, early harvesting, adequate humidity, and removing debris from the preceding harvests in order to reduce proliferation of fungi and prevent mycotoxin production [218,219]. Pre-harvest preventive strategies include favorable storage practices, appropriate environmental factors, good manufacturing practices (GMPs), and good agricultural practices [216]. Good agricultural practices include improving gene modification to suppress production of mycotoxin, analyzing the soil to know the need for fertilizer addition, adequate seed bed treatment, implementing a crop rotation program, and the use of approved fungicides, herbicides, and insecticides for controlling eradication of weeds, infections by fungi, and insect damage [220,221].

While complete exposure avoidance appears impossible, the use of vaccination(s) has also received attention. Vaccinating cattle against AFB1 as a means to reduce the carryover of AFM1 into milk has been studied [222,223,224,225]. When AFB1 was conjugated with Freund’s adjuvant and with a carrier protein, vaccination produced anti-AFB1 antibodies, typically from the IgG class; they reduced the AFM1 carryover into milk for a long time in animals feeding on contaminated feeds [222]. Another subsequent study showed that administration of pre-calving increased the vaccine effectiveness [225]. Fungistatic agents, including weak propionic, benzoic, and sorbic acids, could prevent spoilage, similarly shown via the efficacies of antioxidants, including resveratrol, propylparaben, butyrated hydroxyanisole (BHA), and butylated hydroxytoluene (BHT). Moreover, many antioxidant effects might be synergistic to further prevent mycotoxin production [226,227]. Additionally, using the agents of biological control, including antagonistic fungi, has been known as a significant pre-harvest strategy for preventing contamination of mycotoxins in staple grapes, apples, and cereals [228]. GMPs in food processing plants have to be applied together with GAPs to concomitantly act with HACCPs (hazard analysis critical control points) [228]. Among all the environmental factors, humidity and temperature exert the most significant effects on mycotoxigenic fungal species for mycotoxin production. As it has to do with a favorable storage regimen, moisture, humidity, and temperature of storage rooms/facilities are critical factors for mycotoxin production and mold growth [216].

### 4.2. Post-Harvest Preventive Measures

Stored crops are part of man-made ecology, encapsulating grains, insect pests, rodents, and contaminating mold interacting within environmental conditions such as chemicals, gas composition, availability of water, and temperature [229]. Essential oil usage, including cinnamon oil, clove oil, and bay oil, has shown to prevent some mycotoxin production [226]. The strategies for disinfection, including diatomaceous earth, ozone, or phosphine, are added tactics for the control of insect pests; modified atmosphere storage has been shown to be effective in controlling insects and mold growth in stored grains [227]. Detoxification and decontamination of mycotoxins in several types of agricultural produce is a practical and scientific problem worldwide. Post-harvest, preventing insect damage, processing, transportation, proper storage, drying immediately after harvest, and proper handling are essential [217,218,226,227]. It is recommended to dry crops in an environment with a moisture content below 14% to prevent the accumulation of mycotoxins [226,230]. It has been demonstrated that mycotoxins could be removed using natural methods, including low-temperature plasma, radiation treatment, and thermal insulation, as well as biological methods using biological agents and chemical methods, including absorption, alcoholysis, hydrolysis, reduction, and oxidation [231]. Physical and chemical methods of detoxification have many limitations; they require expensive equipment, are ineffective and time-consuming, and cause nutrient loss [232,233]. Biological methods have been shown to be more environmentally friendly, more specialized, and more effective [234,235]. Post-harvest, using chemical, physical, and/or biological methods and techniques for aflatoxin detoxification have been found to have significant effects.

#### 4.2.1. Biological Strategies

In recent years, many researchers from diverse academic backgrounds have made significant accomplishments in developing biological agents for detoxifying mycotoxins [236]. The use of microorganisms, including fungi and bacteria, to degrade mycotoxins in feed and food has been widely demonstrated [237]. For instance, lactic acid bacteria (LAB) and other species of bacteria, such as *Micrococcus luteus* and *Bacillus subtilis*, can bind with fumonisins B1 and B2, most likely through bacterial peptidoglycan, and probiotics, including *Bifidobacterium* and *Lactobacillus* species [116,238,239]. *Saccharomyces cerevisiae* is among the emerging microorganisms most effective at binding with AFB1, decreasing the latter’s levels in peanuts inoculated with the spores of *Aspergillus parasiticus*. This likely happened either due to the mycotoxin’s adhesion to the cell wall of the yeast or degradation of the mycotoxin [225,240]. Mycotoxin degradation/detoxification using biological strategies provides an alternative approach for mycotoxin control as it can result in the production of little or no end products and/or toxic intermediates. Fermentation effectiveness in mycotoxin elimination and reduction has been shown. However, biological control may not be applicable to some foods and feeds [241]. Some researchers have shown that biological detoxification of mycotoxins and its capacity to change their chemical structures is worthy of increased focus [211]. Some of the organisms used to achieve this include *Flavobacterium aurantiacum*, *Nocardia corynebacterioides*, *Mycobacterium fluoranthenivorans*, *Lactobacillus rhamnosus*, *Saccharomyces cerevisiae*, and *Enterococcus faecium* [211]. To expatiate further and differentiate from [211], we briefly touch on fermentation and dietary diversification, fungi, bacteria, and yeast.

##### Fermentation and Dietary Diversification

Food fermentation enhances the quality of food and at the same time provides specific desirable characteristics that appeal to consumers. A relatively cost-effective approach for mycotoxin disinfection, fermentation could help to eliminate/reduce mycotoxins, to enhance the food ingredients, and to turn foods into more desirable products. However, the fermentation products’ toxicity as well as the nature of its metabolites require careful checks for the sake of food safety [228]. Besides, a typical example of fermentation effectiveness in mycotoxin elimination and reduction is patulin [241]. Most of the major strategies for preventing and controlling mycotoxin exposure include diet diversification, monitoring and surveillance programs aimed at avoiding human exposure, controlling the formation of toxins, and detoxification using chemical, biological, and physical techniques. Strategies to prevent illness caused by mycotoxin exposure are mainly preventive in approach [25]. Besides, the dietary diversification would be a viable approach for controlling the exposure to mycotoxins and may ameliorate the effects of chronic exposures in addition to allowing for the intake of nutrients (or food constituents) that may counteract the mycotoxins [242,243]. Other researchers elsewhere showed that increasing dietary diversity in a Chinese city over a period could bring about reduced aflatoxin exposure and a reduction in liver cancer occurrence [242].

##### Fungi

Control using fungi is another preventive strategy [217]. Some species of fungi, including *Aspergillus niger* and a non-aflatoxin producing strain of *A. flavus*, have the capability to detoxify AFB1 by transforming it into aflatoxicol; when *Rhizopus oligosporus*, a fungus, was cultured alongside AFB1-producing *A. flavus*, it prevented synthesis of AFB1 or stimulated its degradation [28,244]. *Sphingopyxis* species recombinant enzymes can detoxify fumonisins, and these recombinant enzymes can hydrolyze fumonisins B1 to HFB1, where the latter is subsequently deaminated [116]. The application of non-toxic strains of *A. parasiticus* and *A. flavus* on peanuts, pistachios, cotton, and maize recorded noteworthy success in the reduction of aflatoxin contamination [221,228]. Fungi that can produce aflatoxins may also degrade them. This is because during starvation, the fungi have the ability to degrade and possibly convert the products of degradation for use as an energy source. Some fungi, including *Penicillium*, *Clonostachys*, *Trichoderma*, *Rhizopus*, and *Aspergillus* species, can capably and effectively detoxify mycotoxins [221]. In eastern and western Africa, the biological control of aflatoxins in maize with non-toxigenic microbial strains was found to depend on competition. Additionally, reasonable quantities of non-toxigenic inoculants of *A. parasiticus* and *A. flavus* could enter into the soil surrounding the crops, thereby competing with the toxigenic strains [228].

##### Bacteria

Some bacteria in the soil, including *Mycobacterium fluoranthenivorans*, *Nocardia asteroides*, and *Rhodococcus erythropolis* also have the ability to degrade aflatoxins [28,116,238]. Some bacterial species can bind with mycotoxins in food and beverages. *Flavobacterium aurantiacum* B-169 is the only bacterium in over a thousand bacteria tested for possible aflatoxin degradation with the capability of irreversible removal of aflatoxins from solutions. AFB1 detoxification using *Enterococcus faecium* can be achieved via binding to the bacterium cell-wall elements. Polysaccharides and peptidoglycans of the cell walls of bacteria were reported to cause the mycotoxin binding with the microorganisms’ aid [245]. Additionally, DON detoxification with bacteria evolved as a result the advances and efforts in research. Aerobic partitioning and oxidation of DON into C3 carbon achieved with several *Devosia* species offers solutions targeted at decreasing contamination levels of DON [236]. In aqueous solutions, *Lactobacillus reuteri* and *Lactobacillus casei* (lactic acid bacteria) have shown effectiveness in binding with aflatoxins. Other in vitro studies reported that *Lactobacillus rhamnosus* and *Lactobacillus amylovorus* bound to AFB1 with up to 60% efficiency, indicating their ability to bind contaminants in foods. In addition, reductions of 84% of T-2 and 98% of FB1 were reported in whole-grain sorghum fermentation using *Lactobacillus fermentum* [246].

##### Yeast

Using competing yeasts has gained much interest as yeasts produce compounds with antimicrobial properties and have benefits in animals and humans. Additionally, unlike several bacterial antagonists or filamentous fungi, yeasts produce neither secondary metabolites nor allergens [247]. *Saccharomyces cerevisiae* would significantly degrade the DON and decrease the levels of lactate dehydrogenase (LDH), that have been released in cells (stimulated by DON) [248]. Also, low OTA and AFB1 concentrations in chicken diet can be reduced by adding cell walls of *S. cerevisiae* [249]. Patulin reduction using *S. cerevisiae* could be effective when both time and temperature of fermentation are increased. Moreover, yeasts could remove patulin through physical adsorption. Besides, patulin could equally interact with the N-H/O-N polysaccharide and protein bonds of yeast cell walls [241]. Another study reported that *Kluyveromyces marxianus* could bind with OTA, ZEA, and/or AFB1. Generally, mycotoxins able to bind to cell membranes, including those of yeasts such as *C. utilis and Yarrowia lipolytica*, would reduce the OTA concentration by 50% of initial levels in a culture [250].

#### 4.2.2. Physical Strategies

Physical processing has been applied in foods for various purposes [251]. Sorting, removal of affected parts, grading, peeling, microwave heating, extrusion, irradiation, roasting, boiling, milling, segregation, cleaning, washing, and drying are common physical treatments used for the decontamination of mycotoxins [228,252,253]. We briefly touch on storage conditions, radiation (irradiation), use of mycotoxin binders, storing and cleaning, and cold plasma, among other treatments.

##### Storage Conditions

The conditions of storage play a significant role in mycotoxin control as they have an effect on overall fungal growth and activities. High humidity and temperature are two major storage factors that promote the growth of fungi and mycotoxin production. Controlled conditions of storage, including proper air humidity, ventilation, temperature control, and packaging practices, reduce fungal growth and mycotoxin accumulation [254]. About 20–50% of losses in crops were reported in developing nations as a result of inappropriate storage practices [255].

##### Radiation (Irradiation)

The application of natural detoxifying agents like radiation for storage of several cereal grain types is well documented in scientific literature. Radiation can be ionizing or non-ionizing [256]. Radiation can either eliminate or reduce pathogens, but only partly remove or reduce mycotoxins in foods. Radiation can also be applied at an industrial scale [257]. Studies demonstrated that safe levels of about 10 kGy irradiation could decrease the toxicity levels of ZEA-infected tomato, orange, and pineapple fruit juices. However, higher radiation doses would affect the quality of the fruit juices [258]. Elsewhere, 50 kGy irradiation with beam of electron applied in naturally infected corns to degrade OTA and ZEN obtained respective reductions of 67.9% and 71.1%. Additionally, more than 95% AFB1 reduction at 6 kGy would be accomplished after gamma irradiation was applied to the processing of rice [254]. Another study showed the importance of irradiation time, where up to 5 min applied to apple juice could result in about 83% reduction in patulin levels [166]. Despite the capacity of irradiation to reduce the level of mycotoxins in food, it has not yet been the recommended method due to a high chance to produce molecular reactions. Essentially, if irradiation is to be applied to foodstuffs, it must comply to specific standard operating procedures in laboratories already approved by the Food and Drug Administration (FDA) and International Atomic Energy Agency (IAEA) joint committee [211].

##### Use of Mycotoxin Binders

Substances that bind to mycotoxins can inhibit their absorption. This happens because the binding to mycotoxins would prevent their entrance from the gut into the blood. Examples of absorbent materials can include cholesterol, complex indigestible carbohydrates, aluminosilicates, and activated carbon [259]. The use of mycotoxin binders offers an alternative physical technique for aflatoxin degradation [260]. The lactone ring cleavage is a target for enzymes, and this cleavage would decrease the aflatoxins’ level of toxicity [260]. A study showed activated carbon could remove aflatoxins and patulin from naturally infected milk and cider, respectively. Despite the reductions in mycotoxin levels, the study suggested there was need for additional studies to ensure safety and quality [257].

##### Sorting and Cleaning

Sorting and cleaning are among the first-line stages of natural decontamination. Sorting and cleaning would be ideal if they do not pose a risk to increase the number of degraded products [216,252]. Sorting to remove rotten and low-quality fruits and grains has been reported to significantly decrease levels of patulin in fruits/fruit products by nearly 99% [216] as well as total fumonisin B levels by 26% to 69% after maize purification [252]. Sorting has also been reported to reduce between 27 and 93% FB in infected maize. Given the heterogeneity in aflatoxin contamination, separating the damaged nuclei could effectively decrease its contaminations levels. Sorting using ultraviolet radiation could also be used to reduce aflatoxins in cereals [257].

##### Cold Plasma

Cold plasma uses a low-temperature plasma (which can be considered as non-thermal technology), largely produced through the electrical discharge in gases or reduced pressures (subatmospheric pressures). Besides cold plasma needing much less power, it has demonstrated positive effects, particularly in inactivating or preventing fungi’s growth-producing mycotoxins, as well as degrading mycotoxin structures [261]. Plasma could utilize different compounds, which are able to react with biological cells and molecules. The changes arising from such reactions constantly evolve with the inactivation processes, especially at the molecular and morphological levels, which could eventually destroy the mycotoxins [261]. The use of cold plasma has promising antimicrobial properties and its application in food processing aims to eliminate pathogenic microorganisms [256,257,262]. Cold plasma (under low pressure) was also shown to have the potentials to detoxify about 50% of aflatoxins found on the surface of typical nuts [263]. Elsewhere, cold (atmospheric pressure) plasma significantly reduced FB1 and AFB1 in maize by about 66% after 10 min [262]. Plasma treatments for 5 sec resulted in about 100% DON, NIV, and AFB1 degradation. Additionally, exposure to cold (atmospheric) plasma for 8 min resulted in about 93% FU, 100% ZEA, 90% trichothecene, and 93% aflatoxin reductions [264].

##### Other Processing Methods (Frying, Baking, Peeling, Drying, etc.)

Food processing methods have noticeable effects on the properties and compositions of foods [251,265,266,267,268,269,270,271]. Processing techniques decrease mycotoxin concentration but do not completely eliminate them [255]. Softening could reduce mycotoxin contamination levels as fungi gather on the surface of the granules. Peeling, on the other hand, could also help reduce aflatoxins in maize [256]. The maize flour could be less contaminated despite the high levels of ZEN and DON that could occur on the surface of the granules. Moreover, both time and temperature could also influence the levels of mycotoxin in the final (maize) products. Though mycotoxins have thermal stability, several conventional food preparation methods (e.g., frying, baking, roasting, cooking, etc.) at temperatures above 100 °C are likely to also reduce the levels of some mycotoxins. Using extrusion, granules’ moisture content as well as processing temperature influence aflatoxin reduction by 50 to 80% [256]. Additionally, temperatures between 150 and 200 °C would significantly reduce aflatoxin B1 levels at an average decrease of 79% and could be even more effective under increased humidity [272]. In Tanzania, one study employed farmers from three agro-ecological zones in a rural region to study maize and found that drying of the maize on raised a platform or mat; sorting grains for the removal of discolored, moldy, and damaged grains; and using synthetic insecticides while storing grains led to reduced fumonisin and aflatoxin contamination, and these actions were recognized as excellent post-harvest preventive strategies for the prevention of mycotoxin contamination [273].

#### 4.2.3. Detoxification with Enzymes

The detoxification of mycotoxins using enzymes integrates both biological and chemical processing features. Specifically, enzymatic detoxification of mycotoxins requires high specialization, expertise, and performance. It can be considered to occur in mild conditions as it is not toxic to organisms. Additionally, as catalysts, enzymes have a role to play in the non-stoichiometric ratios of mycotoxins [231,256]. Notably, certain species of *Aspergillus* produce enzymes that naturally detoxify fumonisins. The working of such enzymes like chitinase and β-1,3-glucanase against pathogenic microorganisms could differ based on the microbial characteristics [228]. The application of chitinases and β-1,3-glucanases could delay fruit spoilage as well as fungal growth. The inhibition of *A. flavus*, *A. niger*, *Penicillium simplicissimum*, and *P. nalgiovense* complex growth has been shown on samples of salami surface via the spraying of chitinase at concentrations of 40% and 50% and β-glucanase at a concentration of 50% [228]. Chitinase and β-glucanase might be safe alternatives to control fungal spoilage at fermented sausage facilities. Microbial laccase enzymes, catalase, oxidase enzymes, and manganese peroxide can help to detoxify the aflatoxin B1 [228].

#### 4.2.4. Chemical Strategies

##### Chitosan Usage

Chitosan, a derivative from chitin, possesses antioxidant, anticancer, and other non-toxic properties. Chitosan’s antimicrobial properties and biocompatibility make it an interesting biopolymer with wide array of applications [274]. The ability of chitosan to control the growth of fungi hence production of mycotoxins was shown. Specifically, chitosan reduced *F. graminearum* growth, which downregulated the transcript of the major genes that had been involved in the cell growth, respiration, virulence, and trichothecenes biosynthesis. Chitosan would also decrease the fungal spread and mycotoxins accumulation, which demonstrated that the non-toxic chitosan could serve as powerful molecule and potential replacement of the conventional fungicides [274]. Another study showed that applying about 1% chitosan and 1% lemon essential oil could prove to be effective decrease the degree of mycotoxins [275]. Solís-Cruz et al. [276] used an in vitro digestive model that simulated the three gastrointestinal compartments of poultry to evaluate the adsorption capacity of chitosan on aflatoxin B1, fumonisin B1, ochratoxin, trichothecene (T-2), DON, and zearalenone compared with the three cellulosic polymers. These researchers showed that the adsorbent capacity of chitosan obtained much less binding activity against all of the mycotoxins when compared to the three cellulosic polymers used.

Abbasi Pirouz et al. [277] optimized the removal of mycotoxins in palm kernel cake (PKC) using chitosan. Specifically, the mycotoxins studied included aflatoxins (AFB1, AFB2, AFG1 and AFG2), ochratoxin A (OTA), zearalenone, fumonisins (FB1 and FB2) and trichothecenes DON, HT-2 and T-2 toxin. The chitosan achieved a maximum removal of AFB1, AFB2, AFG1, AFG2, OTA, ZEA, FB1, and FB2 at optimized conditions at 94.35, 45.90, 82.11, 84.29, 90.03, 51.30, 90.53 and 90.18%, respectively. Zachetti et al. [278] determined the combined effects of chitosan together with a_w_ on the in situ growth and mycotoxin production in two key *Fusarium* species (*F. proliferatum* and *F. verticillioides*) present in maize, and on *F. graminearum*, the main pathogen-causing *Fusarium* head blight on wheat. Reduction in DON and FB productions via low-molecular-weight chitosans were obtained at the lowest dose (~0.5 mg/g) with more than 70% deacetylation. Whereas decreases in the growth rate of *F. graminearum* occurred at the lowest chitosan dose (~0.5 mg/g), decreases in the growth rates of *F. verticillioides* and *F. proliferatum* occurred at the highest chitosan dose (~2 mg/g).

##### Ozone (O_3_)

Ozone usage in degrading many mycotoxins has been reported [279,280,281]. Ozonation is a common process in many industries and leaves no toxic residue [282,283]. The total applied ozone dosage can be determined by multiplying the O_3_ gas concentration by exposure time and the product divided by the volume of the ozone-processed sample [284]. Ozone can be applied to disinfect vegetables, fruits, and cereals as well as for mycotoxin detoxification [281,285]. Ozone gas was shown to degrade aflatoxins, especially aflatoxins B1 and G1, as their structures have a C_8_–C_9_ double bond [286]. The structural differences in mycotoxins account for how they differ in their responses to ozone. Aflatoxins, for example, comprise hypertoxic sites within the furan ring and that is what the ozone aims to destroy through the creation of primary ozonides [287,288]. The detoxification mechanism for some mycotoxins still remains unclear. Despite this, the oxidizing agents would be able to react with the functional groups within the mycotoxin molecules. The result can be a change in their molecular structures, which allows for the formation of products that have fewer double bonds, molecular weight, and less toxicity [289].

The mechanism of the antifungal property of ozone gas is explained through the damage it does to the fungal membrane [290]. The membrane structure differs across fungal species. This makes some fungal species have more resistance over others specific to ozone treatments. On the one hand, aqueous ozone is able to control fungal growth, while on the other, gaseous O_3_ provides more efficacy to decrease the mycotoxin levels [291,292]. In high-moisture (MC = 21.9%) wheat samples, Wu et al. [293] showed that a high fungal inactivation rate can be achieved at high temperatures (40 °C), the latter able to accelerate the rate at which ozone decomposed into free radicals, considering that ozone decomposes rapidly in water as well as high-moisture products [211]. Treatment with ozone under optimal conditions (55 g ozone per h for 6 h) indicated a reduction in DON by 29 to 32% and in DON-3-glucoside (modified form of DON) by 44%. A significant decline in microorganisms was reported in durum wheat without affecting the rheological and chemical properties of pasta and semolina made from ozonated wheat [280]. Other researchers have shown the capacity of ozone reducing mycotoxins, like the use of aqueous ozone to degrade trichothecene mycotoxins [294], the detoxification of aflatoxin B1 in red peppers [295], as well as the use of gaseous ozone and ozonated water to remove aflatoxin B1 from dried figs [296].

**Figure 4:**
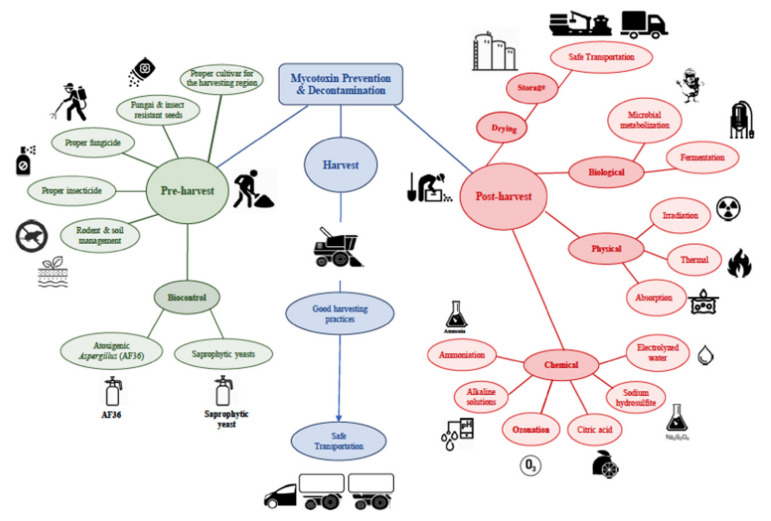
Schematic display of pre- and post-harvest tactics that can be applied to achieve mycotoxin prevention/detoxification, and their links to good harvesting practices and safe transportation (Reprinted/Adapted with permission from Afsah-Hejri, Hajeb, & Ehsani [211], 2020, John Wiley & Sons, Inc).

In a nutshell, considering all above-discussed topics, we can see how common pre- and post-harvest tactics can achieve mycotoxin prevention/detoxification, as shown in Figure 4 and reported by Afsah-Hejri, Hajeb, and Ehsani [211], and their links to good harvesting practices and safe transportation.

##### Bases (Hydrated Oxide, Ammonia)

Seed treatment using ammonia, whilst able to reduce several mycotoxins, such as fumonisins, aflatoxins, and ochratoxins, to non-detectable levels could also be suppressing the mycotoxigenic fungi growth. The application of calcium hydroxide and glycerol mixture could significantly detoxify mycotoxins [216]. Despite potassium hydroxide and sodium hydroxide being commonly applied to achieve AFB1 degradation in contaminated oil, there could still be unwanted and toxic reactions [297]. Bankole [298] studied the effects of ethylene oxide and methyl formate fumigation on seeds’ mycoflora and the germination of some stored oil seeds. This researcher demonstrated that seed fumigation with ethylene oxide and methyl formate would significantly decrease the incidence of fungi, which included the toxigenic species in stored groundnuts and melon seeds. Kavita and Reddy [299] studied the effect of chemicals on aflatoxin B1 production, germination, and viability in maize and groundnuts. These researchers showed that sodium chloride (2.5, 5.0, and 10.0%), propionic acid (1.0, 2.5, and 5.0%), acetic acid (1.0, 2.5, and 5.0%) would inhibit aflatoxin B1 production in *A. flavus*-inoculated groundnuts and maize already kept in gunny bags. These researchers also revealed that all treatments with the exception of sodium chloride could adversely affect both seed germination and viability.

#### 4.2.5. Other Emerging Strategies

##### Nanoparticles (NPs)

NP adsorbents could also be used to remove mycotoxins. As chitosan-coated NPs of Fe_3_O_4_ have been used to decontaminate patulin, magnetic carbon nanocomposites have been used to detoxify AFB1. Additionally, silver NPs have been used to degrade species of *Fusarium* and their mycotoxins [300]. In another study, Lee et al. [301] synthesized a novel photocatalyst nanoparticle called up-conversion NP (UCNP@TiO_2_) and used it for DON degradation. These researchers showed DON reduction in products of cereal less than 1 ppm after 90 min and complete DON degradation after illumination for 120 min. The composite material of UCNP@TiO_2_ was green and efficient, and the products of degradation had little or no toxicity [301]. Additionally, about 87% mycotoxin elimination from mixtures of nanocomposites composed of bentonite, aluminum oxide, and activated carbon has also been reported [302].

##### Extracts from Plants

Several essential oils and their bioactive compounds have been used for their anti-mycotoxigenic and antifungal effects and have been shown to inhibit some mycotoxin production [270,303,304]. Botanical usage is often preferred for the removal of mycotoxins and toxigenic fungi compared to chemical treatments as it is considered environmentally friendly and safe. Clove oil, turmeric oil, and eugenol could also inhibit both AFB1 production and growth of *Aspergillus* species. The application of whole clove in rice grains and culture media could also suppress *Penicillium citrinum* and *Aspergillus flavus* growth and their mycotoxins [305]. Another study investigated the effects of “Pimentón de la Vera” (Spanish paprika smoker) on *P. nordicum* and *A. parasiticus* development and AFG1, OTA, and AFB1 production. It was shown that the addition of between 2 and 3% “Pimentón de la Vera” in meat products, including preparations of sausage and fillets, could reduce the production as well as the development of ochratoxins and aflatoxins [306]. Capsaicin would also inhibit the production of OTA in grapes via *Aspergillus carbonarius* at 61.5%, and via strains of *Aspergillus Nigri* 28.9–78.1% [307].

##### Other Emerging Green Strategies

High-pressure processing (HPP), pulsed electric field (PEF), as well as ultrasound methods have been identified as emerging and green technologies useful in controlling fungi and mycotoxins in foodstuffs [261]. With respect to HPP, the structure of mycotoxins could be altered, which in turn reduces both their toxicity and ability to thrive in the environment. Some other researchers who applied HPP (550 MPa of pressure) to maize grains at a temperature of 45 °C and holding time of 20 min showed that the mycotoxin caused by *F. graminearum* could be reduced substantially [308]. Elsewhere, HPP combined with moderate heating could also deactivate two heat-resistant fungi, *Aliivibrio fischeri* and *Talaromyces macrosporus*, in strawberry puree [309].

PEF demolishes the position of the cell membrane such that a transmembrane voltage gets formed via the assembly of potential diversities between the biofilms of the inside and outside parts, reaching a certain threshold after which permanent cell structural changes occur, then the cell death [261,310]. PEF can be efficacious at conditions of 20–65% output voltage, 10–26 ls pulse width, and a pH range 4–10 to achieve total aflatoxin degradation in a model system [311]. In the shortest possible time, PEF can also offer a peak destruction efficacy of about 99.84% to kill *F. oxysporum* fungi within a nutrient solution, where exposure time plays a great role to achieve disinfection efficiency [312].

With respect to ultrasound, whether it is either low intensity (low energy/power: frequencies ≥100 kHz with lower than 1 W/cm^2^ of intensities) or high intensity (high energy/power: frequencies between 20 and 500 kHz with intensities ≥1 W/cm^2^) it has been shown to decontaminate cereal grain products [261]. An ultrasound frequency 22–35 kHz and intensity from 0.3 to 1.5 W/cm^2^ was shown to decontaminate wheat grain, thereby reducing the fungal content and preventing the formation of mycotoxins [313]. An ultrasound frequency of 40 kHz at a power of 60 W that combined other treatment methods (osmotic dehydration and K_2_CO_3_ emulsion) as a pretreatment of fresh figs (30 min) could significantly mitigate the mycotoxigenic fungal growth and consequently mycotoxin production [314].

## 5. Conclusions and Future Prospects

Mycotoxins affecting animals, foods, humans, and plants specific to types and toxicities as well as strategies employed in their detoxification/removal have been revisited in this paper. The effects of mycotoxins indeed can negatively impact animals, foods, humans, and plants given the considerable variations in toxicities exerted. Tackling mycotoxin contamination of agricultural commodities remains among the key challenges that confront many countries across the globe, which has led to various preventive measures. Indeed, mycotoxin decontamination, control, and detoxification strategies cut across pre-harvest and post-harvest preventive measures. It is very clear that mycotoxin decontamination and detoxification approaches deliver a wide range of outcomes.

Given the pre- and post-harvest mycotoxin challenges associated with the agrofood product industry, more research must be encouraged, from literature synthesis and meta-analyses to analytical/experimental studies, to help supplement existing information. For example, more information is required regarding the degree of damage pre-harvest mycotoxin effects have at the post-harvest stages to animal and plant products. More needs to be done to decipher how mycotoxin prevention measures as well as detoxification/removal strategies during agrofood production, as an example, can be optimized. Future studies could evaluate which specific good harvesting practices, as well as good transportation practices, mitigate mycotoxin toxicities better within the food supply chain and the overall animal–food–human–plant ecosystem. Overall, we consider this contribution herein very useful because it engaged with the animal–human–food–plant aspects of mycotoxins for general public health.

## Data Availability

Data sharing not applicable.
